# An evaluation of artificial intelligence assisted prostate biopsy reporting in the Articulate Pro study

**DOI:** 10.1038/s41746-026-02592-8

**Published:** 2026-05-22

**Authors:** Lisa Browning, Richard T. Colling, Jon Oxley, Jacqueline Birks, Nasullah Khalid Alham, Stefano Malacrino, Chuer Zhang, Abhisek Ghosh, Rosalin Cooper, Monica Dolton, Margaret Horton, Juan A. Retamero, Andrew Protheroe, Leila Bibi Ahmed, Nahida Banu, Anastasios Chatzitolios, Irini Danial, Samir Al-Hyassat, Bidisa Sinha, Pallavi Borkar, Pelvender Gill, Richard J. Bryant, Jonathan Aning, Kieron White, Richard Scheffer, Ewart Stanislaus, James Crofts, David J. Snead, Nasir Rajpoot, Kate Hutton, Davina Hewitt, Clare Dunstan, Rob Procter, Clare Verrill

**Affiliations:** 1 Department of Cellular Pathology, Oxford University Hospitals NHS Foundation Trust, Oxford, UK; 2 Nuffield Departmen of Surgical Sciences, University of Oxford, Oxford, UK; 3 Department of Cellular Pathology, North Bristol NHS Trust, Bristol, UK; 4 Engineering Science, University of Oxford, Oxford, UK; 5 Oxford NIHR BRC, University of Oxford, Oxford, UK; 6 Paige. AI Ltd, New York, NY USA; 7 Department of Oncology, Oxford University Hospitals NHS Foundation Trust, Oxford, UK; 8 Department of Cellular Pathology, University Hospitals Coventry and Warwickshire NHS Trust, Coventry, UK; 9 Bristol Urological Institute, North Bristol NHS Trust, Bristol, UK; 10 Population Health Sciences, Bristol Medical School, University of Bristol, Bristol, UK; 11 Deptartment of Computer Science, University of Warwick, Warwick, UK; 12 Research and Development, University Hospitals Coventry and Warwickshire, Coventry, UK; 13 Alan Turing Institute for Data Science and AI, London, UK

**Keywords:** Diseases, Health care, Medical research

## Abstract

Prospective evidence on clinical utility of AI in histopathology is limited. We conducted a prospective study across three National Health Service specialist centres in England to evaluate a commercially available AI system for assistance in prostate biopsy reporting. Of 1613 cases, 1049 were reported with AI-assistance. Endpoints evaluated diagnostic impact, clinical impact and workflow. Staged AI assistance (second-read) prompted case review and changed the initial diagnosis or Grade Group of 21/386(5.4%) patients, 5 of these (1.3%) potentially affecting clinical management. AI-assisted workflows showed significantly reduced mean turnaround time with concurrent-read compared to unassisted-read by 30.1 h (*p* < 0.0001) at one site with significant reductions in cases requiring immunohistochemistry in all sites (Odds Ratios 0.50,0.43,0.33, *p* < 0.0001, *p* = 0.01, *p* = 0.001). This first prospective, multi-centric evaluation demonstrates AI can enhance diagnostic accuracy, shorten turnaround times and reduce unnecessary testing. Scaled across the NHS, such improvements could improve patient care, deliver faster diagnoses and optimise laboratory efficiency, supporting adoption.

## Introduction

The histopathological diagnosis of prostate cancer is fundamental to clinical management of this malignancy which globally afflicts over 1.4 million new patients per year^1^. In the prostate cancer diagnostic pathway, biopsies are usually acquired via a targeted transrectal or transperineal approach, the tissue is then fixed, stained and prepared for pathologist review. Pathologists assess if malignancy is present and determine the Gleason Score (and Grade Group (GG)) for prostatic acinar adenocarcinoma^2^. The pathology report is later reviewed and discussed in a multidisciplinary team meeting (MDT) to determine whether the patient is offered treatment or active surveillance (AS)^3^. AS involves monitoring localised prostate cancer thus delaying or avoiding radical treatment.

While pathology assessment has been conventionally performed using light microscopy, clinically-approved digital pathology (DP) systems using scanned digital whole slide images (WSIs) in place of glass slides are being increasingly adopted for routine diagnosis^4–6^. DP enables the use of image analysis and artificial intelligence (AI) systems to assist pathologists^7^. There is growing support for both DP and AI in routine clinical use^8^, to alleviate workforce issues and potentially improve diagnostic quality and accuracy^9^. Prostate cancer offers a particularly promising application for AI decision support systems in pathology for many reasons. Gleason Scoring is limited by subjectivity with high interobserver variability^10,11^ and prostate cancer diagnosis rates continue to increase across the global population^12^ amid histopathology workforce shortages^13,14^. AI systems for prostate cancer diagnosis have demonstrated potential to improve interobserver concordance of Gleason grading, reduce diagnostic errors, reduce WSI review time, and reduce use of ancillary tests^15–17^.

Despite the high level of maturity, regulatory approval, and retrospective evidence for AI systems for prostate biopsy reporting, there remain few examples of successful clinical deployment, and a paucity of evidence assessing how AI impacts resources and decisions affecting patients when used real-time in a clinical service. The multi-reader multi-case methodology in the retrospective setting allows lucid comparison of binary human decisions made with and without AI assistance^18^. This approach, however, translates poorly to a fully prospective setting where double reading may not be feasible and could introduce delays in reporting cases. It also does not reflect real-world case-level diagnoses and treatment pathway selection complexity, involving multiple factors beyond binary decisions. Further, it is being increasingly recognised that live clinical evaluations of AI assistive systems should assess impacts on existing care pathways and ecosystems and ascertain human factors^19^. The need for a multifaceted approach beyond traditional in silico evaluation of AI to reflect the complexity of healthcare and human-AI interactions has been highlighted^20^.

To pragmatically address these evidential and methodological gaps, we present the Articulate Pro (Artificial Intelligence for Cellular Pathology Transformation in Prostate Practice) study–a multicentre observational study with the objective to introduce and evaluate AI-assisted tumour detection and grading into standard of care (SoC) prostate biopsy reporting across three UK hospitals. The objectives were to assess how pathology workflows, resource use, diagnostic decisions and user experience are impacted when pathologists use a commercially available AI decision support software device. The technology under evaluation has been previously demonstrated to generalise to different institutions and patient populations^15,21–23^, reduce diagnostic errors, review time, the use of immunohistochemistry (IHC) and second opinions^15,22,24,25^. We assess how AI assistance impacts clinical management, which is not generally addressed in the AI literature. Further, we present a pragmatic approach to validate the clinical and routine use of AI where such guidelines are not yet available.

## Results

### Deployment

**Fig. 1:**
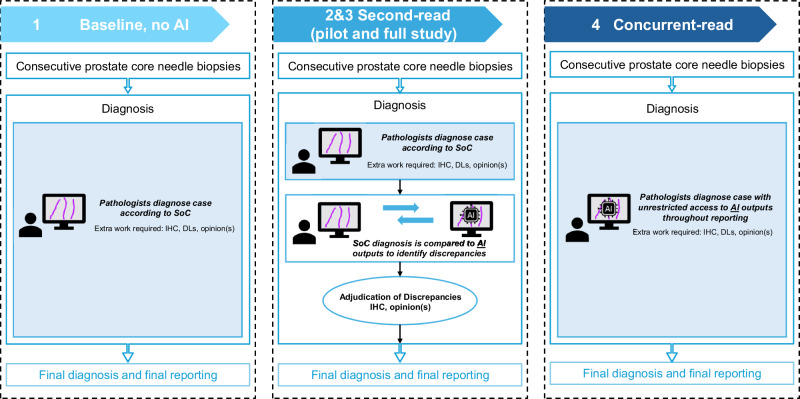
The phases of the Articulate Pro study design. The Articulate Pro study had 4 phases that progressively introduced AI assistance into prostate biopsy reporting. Phase 1 (Baseline, no AI), involved standard of care (SoC) practice to establish a baseline comparator. In Phase 2&3 (second-read pilot and second-read full study respectively), there was staged assistance with AI. Pathologists first diagnosed the case without AI including any extra work such as immunohistochemistry (IHC), deeper levels (DL) or further opinions that might be required. After locking down a report in the study capture tool (SCT) but before formally authorising the case, pathologists accessed the AI outputs. Pathologist-AI discrepancies in case level diagnostic classification or case level maximum Grade Group (GG) according to protocol definitions were recorded and adjudicated, prior to final case authorisation. Phase 4 involved fully AI-assisted reporting, pathologists were able to access AI outputs as desired throughout case review (concurrent read/on-demand use). Figure 1 was created using Microsoft PowerPoint software with the AI icon created using Microsoft Visio software. The prostate biopsy images are illustrated (drawn) using Lucid Spark software.

**Fig. 2:**
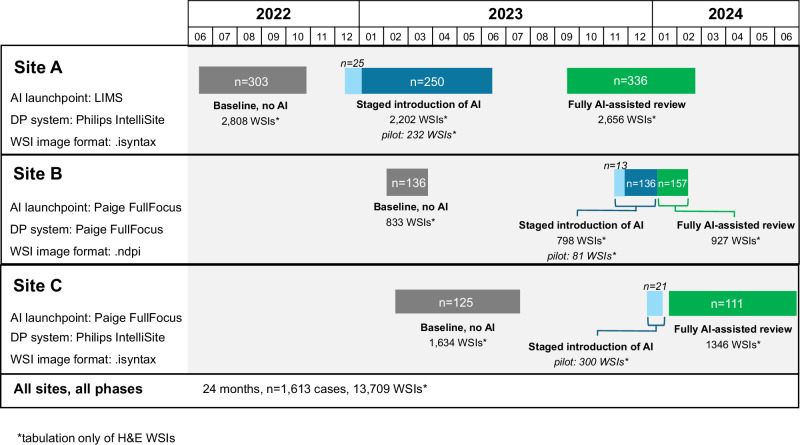
Schedule of Articulate Pro study phases, volumes of cases and whole slide images (WSIs). Measurements were collected over approximately 24 months beginning in June 2022 at site A, followed by sites B and C. Grey indicates the timing duration of Phase 1 of the study (Baseline, no AI), and the coloured bars indicating the study phases involving AI: blue bars are Phase 3 (Staged introduction of AI/second-read, full study), with light blue bars indicating the second-read pilot (Phase 2); green bars are Phase 4 (concurrent/fully AI-assisted review). 1613 cases representing a minimum of approximately 14,000 H&E slides were included in the study across the 3 sites, with 1049 cases (or a minimum of approximately 8500 slides) using AI-assisted diagnosis.

Stepwise deployment and evaluation of the Paige Prostate (PaPr) AI system in prospective NHS workflows was successfully and safely achieved using a phased approach summarised in Fig. 1. 1613 cases representing a minimum of approximately 14,000 Haematoxylin and Eosin (H&E) slides were included in the study across the 3 sites, with 1049 cases (or a minimum of approximately 8500 slides) using AI-assisted diagnosis (Fig. 2). Table 1 provides the population statistics for the cases, including demographic data and case characteristics.

**Table 1:** Population characteristics of the cases included in the Articulate Pro study

	Phase 1			Phase 2			Phase 3			Phase 4		
	Site A	Site B	Site C	Site A	Site B	Site C	Site A	Site B	Site C	Site A	Site B	Site C
	*N* = 303	*N* = 136	*N* = 125	*N* = 25	*N* = 13	*N* = 10	*N* = 250	*N* = 136	*N* = 11	*N* = 336	*N* = 157	*N* = 111
Age												
41–50	8 (2.6)	2 (1.5)	6 (4.8)	0	0	1 (10.0)	2 (0.8)	3 (2.2)	0	7 (2.1)	4 (2.6)	1 (0.9)
51–60	62 (20.5)	26 (19.1)	31 (24.8)	6 (24.0)	4 (30.8)	2 (20.0)	39 (15.6)	25 (18.4)	2 (18.2)	60 (17.9)	45 (28.7)	23 (20.7)
61–70	107 (35.3)	63 (46.3)	54 (43.2)	6 (24.0)	3 (23.1)	3 (30.0)	99 (39.6)	48 (35.3)	6 (54.5)	136 (40.5)	62 (39.5)	51 (46.0)
71–80	106 (35.0)	39 (28.7)	34 (27.2)	13 (52.0)	5 (38.5)	4 (40.0)	100 (40.0)	55 (40.4)	3 (27.3)	118 (35.1)	39 (24.8)	36 (32.4)
80+	20 (6.6)	6 (4.4)	0	0	1 (7.7)	0	10 (4.0)	5 (3.7)	0	15 (4.5)	7 (4.5)	0
Diagnosis												
Benign/PIN	54 (17.8)	35 (25.7)	43 (34.4)	3 (12.0)	3 (23.1)	2 (20.0)	39 (15.6)	26 (19.1)	1 (9.1)	66 (19.6)	34 (21.7)	26 (23.4)
ASAP	18 (5.9)	4 (2.9)	6 (4.8)	0	0	1 (10.0)	23 (9.2)	5 (3.7)	1 (9.1)	13 (3.9)	5 (3.2)	9 (8.1)
Adenocarcinoma	231 (76.2)	97 (71.3)	76 (60.8)	22 (88.0)	10 (76.9)	7 (70.0)	188 (75.2)	105 (77.2)	9 (81.8)	257 (76.5)	118 (75.2)	76 (68.5)
PIRADS score												
1	2 (0.7)	0	3 (2.4)	0	0	0	1 (0.4)	0	0	2 (0.6)	0	0
2	8 (2.6)	6 (4.4)	31 (24.8)	0	2 (15.4)	3 (30.0)	8 (3.2)	6 (4.4)	0	8 (2.4)	5 (3.2)	20 (18.0)
3	44 (14.5)	38 (27.9)	22 (17.6)	1 (4.0)	2 (15.4)	0	27 (10.8)	27 (19.8)	0	25 (7.4)	49 (31.2)	9 (8.1)
4	91 (30.0)	33 (24.3)	24 (19.2)	8 (32.0)	4 (30.8)	2 (20.0)	67 (26.8)	24 (17.6)	2 (18.2)	87 (25.9)	31 (19.8)	25 (22.5)
5	123 (40.6)	25 (18.4)	24 (19.2)	10 (40.0)	4 (30.8)	3 (30.0)	112 (44.8)	30 (22.1)	6 (54.5)	167 (49.7)	40 (25.5)	32 (28.8)
Not stated	27 (8.9)	32 (23.5)	18 (14.4)	0	1 (7.7)	2 (20.0)	24 (9.6)	43 (31.6)	3 (27.3)	29 (8.6)	29 (18.5)	23 (20.7)
X (not performed or technically inadequate)	8 (2.6)	2 (1.5)	3 (2.4)	6 (24.0)	0	0	11 (4.4)	6 (4.4)	0	18 (5.4)	3 (1.9)	2 (1.8)
Type of biopsy												
LATP	154 (50.8)	136 (100.0)	65 (52.0)	11 (44.0)	13 (100.0)	9 (90.0)	98 (39.2)	135 (99.3)	9 (81.8)	110 (32.7)	157 (100.0)	96 (86.5)
TRUS or templated	149 (49.2)	0	60 (48.0)	14 (56.0)	0	1 (10.0)	153 (60.8)	1 (0.7)	2 (18.2)	226 (67.3)	0	15 (13.5)

Grade Group total numbers	*N* = 231	*N* = 97	*N* = 76	*N* = 22	*N* = 10	*N* = 7	*N* = 188	*N* = 105	*N* = 9	*N* = 257	*N* = 118	*N* = 76
Grade Group												
1	23 (10.0)	29 (29.9)	10 (13.2)	5 (22.7)	0	2 (28.6)	29 (15.4)	17 (16.2)	2 (22.2)	27 (10.5)	32 (27.1)	8 (10.5)
2	110 (47.6)	40 (41.2)	31 (40.8)	7 (31.8)	6 (60.0)	1 (14.3)	85 (45.2)	36 (34.3)	1 (11.1)	135 (52.5)	54 (45.8)	32 (42.1)
3	54 (23.4)	16 (16.5)	14 (18.4)	0	2 (20.0)	1 (14.3)	32 (17.0)	26 (24.8)	0	36 (14.0)	19 (16.1)	9 (11.8)
4	17 (7.4)	2 (2.1)	3 (4.0)	5 (22.7)	0	1 (14.3)	20 (10.6)	17 (16.2)	2 (22.2)	23 (8.9)	4 (3.4)	8 (10.5)
5	26 (11.3)	7 (7.2)	16 (21.1)	5 (22.7)	2 (20.0)	2 (28.6)	19 (10.1)	6 (5.7)	4 (44.4)	35 (13.6)	7 (5.9)	17 (22.4)
Not graded (hormone therapy, brachytherapy)	1 (0.4)	3 (3.1)	0	0	0	0	3 (1.6)	3 (2.9)	0	0	2 (1.7)	1 (1.3)
Missing values	0	0	2 (2.6)	0	0	0	0	0	0	1 (0.4)	0	1 (1.3)

Data included age, diagnosis, MRI based PIRADS score, type of biopsy protocol and Grade Group (GG). Absolute numbers shown together with percentages in brackets.

There are often inherent inter lab variations in prostate biopsy reporting practice which need to be taken into account when evaluating impacts of AI. Therefore, a pre-baseline audit, before phase 1 (baseline), was undertaken of the prostate biopsy reporting practices at the 3 centres prior to commencement of study data collection. Prostate biopsies between 1st January 2021 and 31st December 2021 were included, (Supplemental Table 1). There was variation in proportion of malignant diagnoses ranging from 55 to 76%.

Cases were consecutive where possible to maintain clinical services and required successful AI reads without technical issues. 47 cases were recorded as being completely excluded (registered as excluded in the study capture tool (SCT) due to a fundamental issue with including the case in the study, for example due to a duplicate case entry or a technical issue (Supplemental Table 2). 14 of the 47 cases were for AI-platform related reasons (AI not available, bridge not available or slides/cases not uploaded or missing). Excluded cases for an AI platform specific reason comprised 14/1660 total cases (included or excluded) (0.8%) and overall 47/1660 (2.8%) cases were excluded for any reason. Non-AI platform related reasons for exclusion were data entry issue eg duplicate record (*n* = 19), testing phase (case used as a test) (*n* = 7) and other (*n* = 7), e.g., case reported by a non-study pathologist (locum) or non-prostate biopsy. Other cases had issues that were able to be resolved and cases were included in the study, such as needing a rescan.

### AI impact on diagnosis

Comparison of diagnostic categories (cancer/Atypical Small Acinar Proliferation (ASAP) or benign/PIN) between phase 4 (concurrent-read/full AI assistance) and phase 1 (baseline – no AI) is shown in Supplemental Table 3a–c. Reassuringly, there was significant association between phases 1 and 4 at sites A, B and C respectively representing no major diagnostic shifts with AI deployment (*p* = 0.43, *p* = 0.71, *p* = 0.14 respectively). There were no major changes to pathology processes or reporting between phases 1 and 4 in the centres.

386 cases were included in phase 3, which was conducted in full at sites A and B only. It was not possible to conduct phase 3 at site C due to logistical challenges. Site C opened later than sites A and B to the study (Fig. 2) and although it was intended to conduct phase 3, only 11 cases could be reported in this way. This was due to the increased time requirement for the detailed data entry in this phase and mandated adjudication which proved to be a barrier to the study proceeding. The decision was made to truncate the phase and consider it an extended second-read pilot (all part of phase 2) as there was too little data to meaningfully analyse and instead concentrate efforts on phase 4 in order to complete the study. Phase 3 data is therefore not presented for site C. The focus in phase 3 was on impact on diagnostic practice and not turnaround time (TAT) and a full set of data from phase 1 and phase 4 was needed to enable the ‘before and after AI’ comparison, hence why this decision was made. The second-read (staged assistance) protocol of Phase 3 allowed direct comparison of the pathologist’s diagnosis made without AI, compared to the final diagnosis made by the pathologist after viewing and adjudicating AI outputs. Among *n* = 386 cases, the use of AI and adjudication led to case-level changes to *n* = 21 cases (21/386, 5.4%; Site A: 12/250 (4.8%); Site B: 9/136 (6.6%)) (Table 2).

**Fig. 3:**
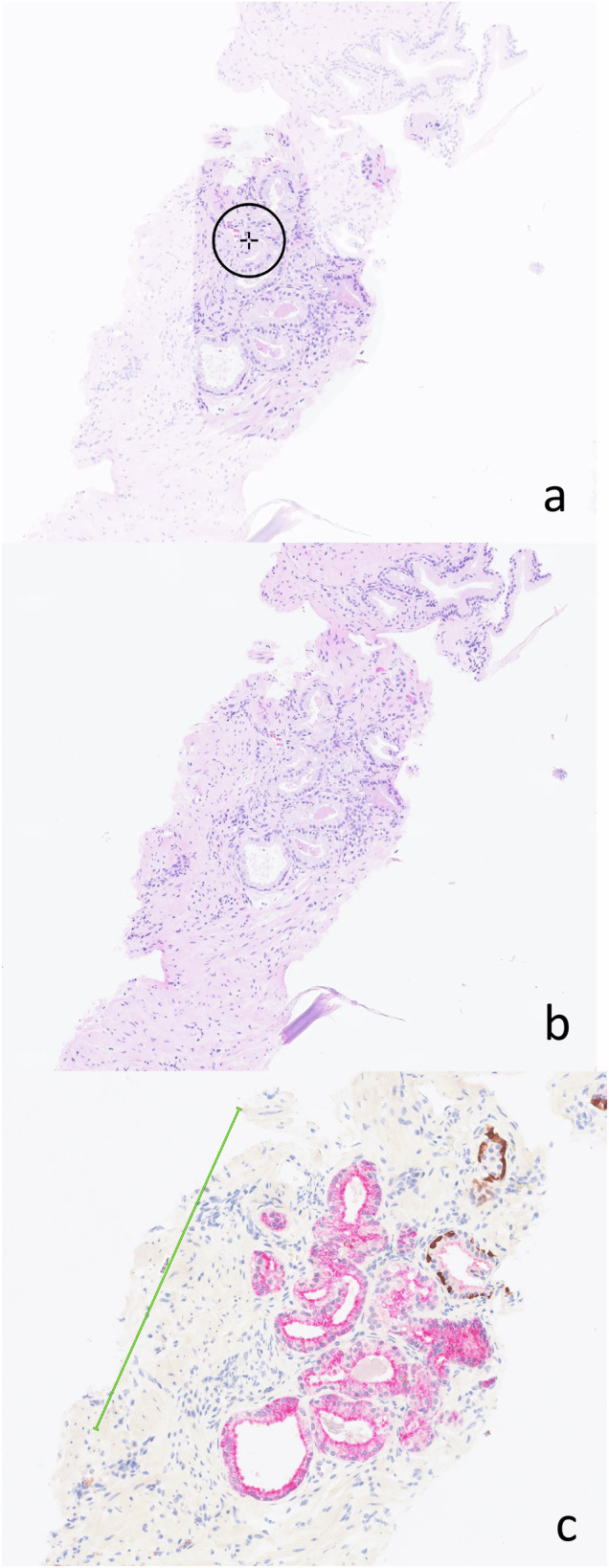
Case changed to Grade Group (GG) 1 adenocarcinoma after AI assistance. The figure shows example histological images of a case originally intended to be signed out as benign but identified by AI as being suspicious of malignancy, with the area on the slide most suspicious of malignancy being highlighted (**a**). The pathologist had seen this focus, but reported a change in threshold due to the reassurance that an AI second-read was due to follow and provide reassurance of the need for immunohistochemistry (IHC). It was adjudicated to final sign out as a focus of GG1, Gleason Score 6 (3 + 3) prostatic adenocarcinoma. Green scale bar is 0.5 mm. H&E (**b**) shown together with P63/AMACR ‘cocktail’ stain (**c**).

Local Discrepancy Boards at sites A and B interpreted in a multidisciplinary setting any possible impact on clinical management from the use of AI assistance in the final report compared to the pathologist non-assisted intended report. 5 changes (5/386, 1.3%) could have been of clinical significance to the patient pathway, while the other case-level changes would not have modified patient treatment pathways (Table 2). 90.5% (19/21) of the case-level changes were to the GG, and there were 2 diagnostic changes one to adenocarcinoma with Gleason Score 6 (3 + 3) GG 1 disease (Fig. 3) which led to the patient being placed on AS and a further case to ASAP (Supplemental Fig. 1) which resulted in ongoing Prostate Specific Antigen (PSA) surveillance. Although a single example, the case changed to GG1 highlights how AI may change the interaction between the pathologist and the case when they know they have the AI review available. That focus had been seen by the pathologist, however on reflection they felt that although they favoured that the tiny focus was benign they had been considering requesting IHC to support this and furthermore anticipated that the AI output would support the need for IHC by identifying the focus as suspicious, which it did. Anecdotally, their actions had been altered by the availability of the AI, illustrating the potential learning curve element of working in conjunction with these tools.

**Table 2:** Cases with a change to the final authorised report after pathologists viewed and adjudicated AI outputs

Case reference	Discrepancy G = Gleason D = overall diagnosis	Grade Group assigned prior to viewing AI outputs	Grade Group output of AI	Grade Group from second opinion adjudication	Final authorised Grade Group	Possible that pre-AI diagnosis would have led to different management?	Possible impact on management in terms of surveillance and treatment options offered to patient	Final management option selected
A_1	G	3	2	2	2	No	n/a	Hormones and radiotherapy
A_2	G	3	4	2	2	No	n/a	Hormones and radiotherapy
A_3	G	3	2	2	2	**Yes**	Active surveillance (AS) was offered as an option for GG2, but would not have been offered for GG3	Hormones and radiotherapy
A_4	G	5	4	4	3	No	n/a	Hormones and radiotherapy
A_5	G	3	4	5	5	No	n/a	Hormones and radiotherapy
A_6	G	3	4	4	4	**Yes**	Treatment more likely than Watchful Waiting (WW) at GG4 than GG3	Hormones and radiotherapy
A_7	G	3	5	4	4	No	n/a	Hormones and radiotherapy and hormone therapy plus (enzalutamide)
A_8	G	2	4	3	3	**Yes**	AS may have been offered for GG2, but was not offered for GG3	Radical prostatectomy
A_9	G	3	4	5	5	No	n/a	Radical prostatectomy
A_10	G	3	4	4	4	No	n/a	Hormones and radiotherapy and hormone therapy plus (enzalutamide)
A_11	G	3	4	4	4	No	n/a	Radical prostatectomy
A_12	G	2	5	3	3	No	n/a	Hormones and radiotherapy
B_1	G	3	5	4	4	No	n/a	Radical prostatectomy, robotic-assisted laparoscopic radical prostatectomy (RALP)
B_2	G	3	4	4	4	No	n/a	Radical prostatectomy (RALP)
B_3	G	4	4	3	3	No	n/a	Hormone therapy
B_4	G	2	3	3	3	No	n/a	Bone scan positive, hormones
B_5	D	n/a	1	1	1	**Yes**	AS was offered and would not have been for a benign diagnosis	AS
B_6	D	n/a	n/a	n/a	n/a - changed to ASAP	**Yes**	If PSA levels were to rise, there will be lower threshold to re-biopsy	PSA monitoring
B_7	G	3	2	2	2	No	n/a	Hormone therapy
B_8	G	3	4	2	2	No	n/a	Radical prostatectomy (RALP), not suitable for radiotherapy
B_9	G	2	3	3	3	No	n/a	Hormones and radiotherapy

Table 2. Cases with a change to the final authorised report as a result of AI assistance (second-read) and adjudication in phase 3. The table shows cases that were adjudicated to a different GG or diagnosis (ASAP/cancer versus benign) in the final authorised report compared to the pathologist initial report that they locked down in the study capture tool (SCT) prior to viewing the AI compared to the final authorised report after viewing the AI and adjudicating outputs. Among *n* = 386 cases, the use of AI and adjudication led to case-level changes to *n* = 21 cases (21/386, 5.4%; Site A: 12/250 (4.8%); Site B: 9/136 (6.6%). Local Discrepancy Boards at sites A and B interpreted in a multidisciplinary setting any possible impact on clinical management from the use of AI assistance in the final report compared to the pathologist non-assisted intended report. 5 changes (5/386, 1.3%) could have been of clinical significance to the patient pathway, while the other case-level changes would not have modified patient treatment pathways.

There were 3 cases with a change to the final authorised GG after a discordance between pathologist and PaPr was adjudicated, which may have impacted patient management. In one case AS was able to be added to management options, but the patient opted for radical treatment; in another case radical treatment was more likely to be offered than watchful waiting; and in a further case AS was not offered due to the change in GG and the patient received treatment.

36.4% (91/250) cases at site A were adjudicated, and 37.5% (51/136) at site B for protocol driven reasons. Non mandated adjudication involved 14/250 (5.6%) of cases at site A, 2/136 (1.5%) of cases at site B. There were 4 cases that were GG discrepancies according to protocol definitions though were not adjudicated and noted in analysis as protocol deviations, the reasons for this being unclear. 9 cases at each of sites A and B in phase 3 were GG4 versus GG5 discrepancies that did not require adjudication, 9/250 (3.6%) site A, 9/136 (6.6%) site B.

### PaPr detect standalone outputs

Standalone PaPr sensitivity and specificity in phase 3 (second-read/staged AI assistance) and phase 4 (concurrent read/fully AI assisted workflow) of the AI at a case level is shown in Table 3. Case level more closely replicates how pathologists report than slide level. The reference is slightly different in each, with mandatory adjudication in phase 3 of discrepant but not all cases and no mandatory adjudication in phase 4. Reference standard was final authorised diagnosis (pathologist plus AI). Phase 3 was not possible for logistical reasons in site C. NPV (76.7–83.3%) at case level during phase 3 seems lower than reported in Phase 4 (86.7–100.0%), and in other studies. Of the false negative cases in phase 3, 9/11 were ASAP rather than adenocarcinoma and in phase 4, 6/6 false negatives were all ASAP. ASAP is a category which lacks clear criteria and shows poor agreement, for example, in this study rates vary across phases and sites from 2.9% to 9.2% cases. In site A, ASAP accounted for 9.2% cases phase 3, 3.9% phase 4, 6.0% phase 1 which may be random differences, or perhaps a feature of the design of phase 3 where pathologists may have looked more carefully, over-thinking the case to avoid AI finding differences once unveiled. The relatively low NPV contrasts with the pathologists’ survey opinion that they felt the most useful AI output was a negative result. Specificity in phase 3 ranged from 59.0 to 76.9% which was lower than phase 4 (82.4–100.00%). A higher rate of false positives was seen in phase 3 than phase 4 which supports the specificity variation, however, case numbers in the benign category in phase 3 are much lower than phase 4 and much fewer than malignant cases in general which more likely accounts for the variation as opposed to a learning curve effect. Confidence intervals for specificity are not so dissimilar for phases 3 and 4.

**Table 3:** Standalone Paige Prostate (PaPr) sensitivity and specificity in phase 3 (second-read) and phase 4 (concurrent-read) of the AI at a case (not slide) level

	SITE A	SITE B	SITE C
**Phase 3**			
Sensitivity	204/211 (96.7%) 95% CI (93.3% to 98.7%)	106/110 (96.4%) 95% CI (91.0% to 99.0%)	
Specificity	23/39 (59.0%) 95% CI (42.1% to 74.4%)	20/26 (76.9%) 95% CI (56.4% to 91.0%)	
PPV	204/220 (92.7%) 95% CI (88.4% to 95.8%)	106/112 (94.6%) 95% CI (88.7% to 98.0%)	
NPV	23/30 (76.7%) 95% CI (57.7% to 90.1%)	20/24 (83.3%) 95% CI (62.6% to 95.3%)	
False positives	16/39 (41.0%)	6/26 (23.1%)	
False negatives	7/211 (3.3%) all ASAP	4/107 (3.7%) 2 ASAP 2 adenocarcinoma	
**Phase 4**			
Sensitivity	267/269 (99.3%) 95% CI (97.3% to 99.9%)	123/123 (100%) 95% CI (97.0% to 100%)	81/85 (95.3%) 95% CI (88.4% to 98.7%)
Specificity	60/67 (89.6%) 95%CI(79.7% to 95.7%)	28/34 (82.4%) 95% CI (65.5% to 93.2%)	26/26 (100%) 95% CI (86.8% to 100%)
PPV	267/274 (97.4%) 95% CI (94.8% to 99.0%)	123/129 (95.3%) 95% CI (90.2% to 98.3%)	81/81 (100%) 95% CI (95.5% to 100%)
NPV	60/62 (96.8%) 95% CI (88.8% to (99.6%)	28/28 (100%) 95% CI (87.7% to 100%)	26/30 (86.7%) 95% CI (69.3% to 96.2%)
False positives	7/67 (10.4%)	6/34 (17.6%)	0/26 (0%)
False negatives	2/269 (0.7%), all ASAP	0/123 (0%)	4/85 (4.7%), all ASAP

Reference standard was final authorised diagnosis (pathologist plus AI). There was mandatory adjudication in phase 3 of discrepant but not all cases and no mandatory adjudication in phase 4. Phase 3 was not possible for logistical reasons in site C. Specificity (59.0–76.9%) and NPV (76.7–83.3%) at case level during Phase 3 seem lower than those reported in Phase 4, and in other studies, but potential explanations for this are discussed in the text. The relatively low NPV contrasts with the pathologists’ survey opinion that they felt the most useful AI output being a negative result.

### Human factors

A total of 72 pathologist survey responses (multiple responses at various time points per pathologist) were received across the duration of the study. The end of study survey is the main focus of the results presented here as a summary of pathologist opinion on the experience using AI in this setting. The response rate for this study was 73% (8/11 pathologists), with all surveys completed in full.

Data from the baseline/pre-study survey, completed by 6 consultant-level study pathologists, revealed that 83% (5/6) had >16 years of experience as a pathologist (including training), with 17% (1/6) having 5–10 years of experience. All reported an estimated 11–30 prostate biopsies per month.

At the end of the study (8 survey respondents), all confirmed having reported at least 50 prostate biopsies with AI assistance. There was 100% (8/8) agreement between pathologists that they felt confident in using the AI noting that this was a generally quite experienced group of pathologists. Level of experience will be covered in more detail in the dedicated survey paper.

All agreed that they were comfortable reviewing the AI output on the whole, although specifically in relation to the review of AI output related to Gleason Score whilst 63% (5/8) agreed they found this easy to interpret, 25% (2/8) disagreed, and 13% (1/8) remained neutral. This reflects the reported subjective confidence that our study pathologists had in the PaPr Detect for harbouring malignancy or appropriately suspicious foci, and in the suggested Gleason Score.

Considering the usability of the AI in their own practice, ‘supporting a diagnosis of benign when no suspicious flags assigned (by the AI)’ was ranked as the most useful outcome, with ‘supporting the presence of malignancy’ ranked second. By the end of the study 100% of the pathologists agreed that for the majority of the cases they felt confident that an AI output with no areas identified as suspicious would, on pathologist review, be benign or PIN at the most, and similarly 100% (8/8) agreed that suspicious foci were not generally missed by the AI.

Free text comments related to the ‘most useful function of the AI’ were as follows;

- *(It is) reassuring if benign and (for) quick overview of malignant case*
- *Supporting pathologist interpretation as benign or malignant*

Considering the future utility of AI in their own practice, all pathologists agreed they would use AI if it were available, with 87% (7/8) agreeing they would use it for reporting *all* (as opposed to *most*) cases (1/8).

In view of the recognised importance of human factors assessment in the evaluation of AI^19^ and in order to capture the comprehensive evaluation we have undertaken during this study, the full survey results will form the basis of a dedicated manuscript to follow. An in-depth qualitative study of pathologists’ experiences of using PaPr using previously described methods^26^ is in preparation in addition to previous publication of preliminary findings on pathologists’ experiences of using the PaPr system^27^. Other clinician and patient views have been previous published^28^.

### Gleason Scoring/GGs

Supplemental Tables 4a–c shows a comparison of GGs between phase 4 after full AI deployment with a concurrent-read and phase 1 (baseline, no AI). There was significant association between phases and thus no statistically significant difference between the phases in the pattern of GG assignment at any of sites A, B or C (*p* = 0.12, *p* = 0.9, *p* = 0.46 respectively).

**Table 4:** Turnaround time (TAT) for cases in phase 4 (concurrent AI read) versus phase 1 (baseline, no AI)

	Phase 1			Phase 4			
Variable and SITE	No. of Cases	MEAN Phase 1	SD Phase 1	No. of cases	MEAN Phase 4	SD Phase 4	Mean adjusted change phase 4 – phase 1
**Total Turnaround Time (TAT) (hours)**							
SITE A	303	163.4	79.2	336	130.6	62.1	-30.1 (95% CI -40.7 to -19.5, *p* < 0.0001)
SITE B	136	134.4	61.9	157	140.1	63.2	6.4 (95% CI -7.8 to 20.6, p=0.4)
SITE C	122	302.7	130.1	108	274.3	108.9	-28.9 (95% CI -61.1 to 3.4, p=0.08)
**Pathologist TAT (hours)**							
SITE A	303	97.4	76.5	336	70.0	55.9	-24.7 (95% CI -34.8 to -14.5, p<0.0001)

Total TAT for all 3 sites is shown with pathologist only TAT shown for site A (data not available for the other sites). Total TAT was the time from receipt in the lab to case authorisation by the pathologist. Pathologist TAT was booking the case out from the lab to pathologist to case authorisation by the pathologist. In site A there was a reduction in total TAT, mean adjusted change −30.1 h (95% CI −40.7 to −19.5, *p* < 0.0001), and this was supported by a reduction in pathologist TAT, mean adjusted change -24.7 h (95% CI −34.8 to −14.5, *p* < 0.0001), phase 4 compared with phase 1. Statistically significant differences were not seen in sites B or C.

In phase 3 (second-read), the case level unassisted pathologist GG together with the PaPr GG data is shown in Supplemental Table 5a, b for sites A and B (not available for site C). Site A showed agreement on GG for 117/183 (agreement percentage 63.2%), kappa 0.51 (SE 0.04) of malignant cases, site B showed agreement for 50/98 (agreement percentage 51.0%), kappa 0.38 (SE 0.05). The level of agreement would be higher if GG4 and GG5 were considered as one category rather than reporting them separately. 13/183 malignant cases (7.1%) were more than one GG different between PaPr and pathologist in site A and 13/98 (13.3%) in site B.

**Table 5:** Immunohistochemistry (IHC) requests comparing phase 4 (full AI assistance with concurrent-read) to phase 1 (baseline/no AI)

	Phase 1	Phase 4	
SITE	Number cases with IHC request/number cases	Number cases with IHC request/number cases	Adjusted odds ratio comparing phase 4 with phase 1 (95% CI)
SITE A	118/303 (38.9%)	81/336 (24.1%)	0.50 (0.35, 0.71) p<0.0001
SITE B	35/136 (25.7%)	22/157 (14.0%)	0.43 (0.23, 0.82) p=0.01
SITE C	50/125 (40.0%)	23/111 (20.7%)	0.33 (0.17, 0.62) p=0.001

It shows a statistically significant reduction in the number of cases requiring IHC in phase 4 compared to phase 1 sites A, B and C, after adjustment for the diagnostic case mix (OR site A 0.5, site B 0.43, site C 0.33, *p* < 0.0001, 0.01, 0.001 respectively). A case with an ASAP diagnosis is far more likely to require IHC, and therefore variation in the number of ASAP cases in the two phases has been taken into account in the calculation of Odds Ratios (OR).

From the survey, by the end of the study 75% (6/8) agreed that the AI output for Gleason grading was in line with their own opinion for most of their cases. With AI experience to hand at the end of the study, the pathologists were divided in relation to their concern that the AI output might influence their own opinion on Gleason grading, with 37.5% (3/8) agreeing and an equal number (3/8) disagreeing on this point (25%, 2/8 remaining neutral). It is therefore important to show as above that there was no apparent grade shift with the implementation of AI.

### Impact of AI on workflow

One primary objective of Articulate Pro was to evaluate the resource use in the current SoC (i.e., there being no AI assistance) compared against a fully AI-assisted workflow, and thus phases 1 and 4 were compared. Equivalency in diagnostic categories and GGs between phase 1 and phase 4 has been shown as above. In sites A and B, the pathologists were the same personnel as between phase 1 and phase 4. In site C, in phase 4, 2/4 pathologists were the same as phase 1. 1 pathologist only took part in phase 1, and 2 pathologists only took part in phase 4.

TAT was defined as the time and date from receipt of the case by the lab to time and date that the case was authorised by the pathologist and is impacted by various factors including IHC requesting, slide viewing and further opinions. TAT with the pathologist only at site A from the Laboratory Information Management System (LIMS) was also analysed to confirm that time in the lab was not responsible for any differences between phase 1 and phase 4 (data only available for site A). Although this data was not available for sites B and C from the LIMS, it is shown for all 3 sites in Supplemental Fig. 2 as box plots of TAT and case activity (from timers in the SCT) showing the distribution of activities and separately by case complexity and by diagnosis. In site A, there is a reduction in pathologist activity related to cases in phase 4 compared to phase 1, particularly in ASAP and more complex cases which mirrors that of the TAT, highlighting a reduction in pathologist activity on cases. The data for sites B and C are more complex and a statistically significant reduction in TAT was not seen at those sites. The total TAT was calculated from the LIMS where available – sites A and B or the SCT (site C) where not available from the LIMS. SCT data was also used to verify TAT for site A. 3 site C cases were excluded (all phase 1) from TAT analysis due to incomplete data.

In site A there was a statistically significant reduction in total TAT, mean adjusted change −30.1 h (95% CI −40.7 to −19.5, *p* < 0.0001), supported by a reduction in pathologist TAT, mean adjusted change −24.7 h (95% CI −34.8 to −14.5, *p* < 0.0001), phase 4 compared with phase 1 (Table 4). In site B there was no statistically significant difference in total TAT, mean adjusted change +6.4 h (95% CI −7.8 to 20.6, *p* = 0.4). In site C there was a similar reduction to site A in total TAT, but it was not statistically significant, mean adjusted change −28.9 h (95% CI −61.1 to 3.4, *p* = 0.08). A breakdown of TAT by pathologist is shown in Supplemental Table 6. In site A which showed a statistically significant reduction in overall mean TAT, all 4 pathologists also showed a reduction in TAT. In site C, where a reduction was seen that was not statistically significant, a reduction is seen for pathologist 1, but not pathologist 2.

The percentage reduction in the number of cases with IHC requests was statistically significant for phase 4 compared with phase 1, after adjustment for the diagnostic case mix (OR site A 0.5, site B 0.43, site C 0.33, *p* < 0.0001, 0.01, 0.001 respectively), shown in Table 5. A case with an ASAP diagnosis is far more likely to require IHC, and therefore variation in the number of ASAP cases in the two phases has been taken into account in the calculation of Odds Ratios (OR). Mean number of IHCs per case that were performed is shown in Supplemental Table 7 and that summary descriptive data shows similar patterns of reduction in phase 4 compared to phase 1.

Further opinion data could only be analysed for site A as it was not routinely recorded at site C for operational reasons and site B operates a routine re-report ‘’in-line MDT review” for all cases. In phase 1 site A, 25/303 (8.3%) cases had at least one further pathologist opinion. In phase 4, 19/336 cases (5.7%) had at least one further opinion, odds ratio 0.66 CI 0.34, 1.26 (*p* = 0.21). Low numbers meant that statistical significance was not able to be shown in relation to further opinions but there was a reduction in the rate, which a larger study may be able to definitively capture.

Considering the impact of AI on workflow, in the pathologist end of study survey there was good agreement that the pathologists could see potential for AI to both save time in reporting cases and improve diagnostic efficiency in this setting, with 75% (6/8) and 88% (7/8) agreeing or strongly agreeing to these points. Whilst we have demonstrated a reduction in IHC requests that was statistically significant at all sites, when pathologists were asked in the survey if AI might reduce the number of requests for immunohistochemistry, only 12% (1/8) agreed that it might, with 75% (6/8) remaining neutral on this point. In relation to whether the use of the AI might increase the number of requests for further opinions from pathologist colleagues, particularly in cases where there was a mismatch between the AI output and the opinion of the reporting pathologist, whilst 12% (1/8) agreed that this might happen, 50% (4/8) disagreed, with the remaining 3 pathologists neutral.

## Discussion

This study represents the first prospective, multi-centre evaluation of an AI system for diagnostic decision support in prostate cancer histopathology within the NHS, with over 1000 biopsy cases reported using AI assistance across three hospitals by uropathology subspecialists. By evaluating not only diagnostic and clinical impact but also real-world workflow and human factors, we address a critical gap in the pathology AI literature, which has been highlighted before^19^. Previous work has focused largely on retrospective datasets or controlled reader studies, which, while necessary, are insufficient to inform implementation in routine practice^22,24,25^. In this regard, a hierarchy for assessing the efficacy of AI in histopathology proposes a six-level approach encompassing technical efficacy (level 1), diagnostic accuracy (level 2), diagnostic thinking efficacy–impact of the software on diagnosis (level 3) and therapeutic efficacy–impact on patient management decisions (level 4)^29^ adapted from an imaging based model^30^. Our work addresses levels 1 to 4 in this hierarchy, extending the current literature significantly beyond merely retrospective studies. Levels 5 and 6 (patient outcomes and societal efficacy) are beyond the scope of this current paper. However, a cost effectiveness/economic analysis of implementing this technology has been conducted and will be the focus of a follow-on manuscript led by York Health Economics Consortium.

This study addresses the complexity of evaluating AI in real-life histopathology workflows, highlighting challenges such as site-specific practices and proposing relevant impact parameters^20^. Building on the framework of eight implementation outcomes (appropriateness, acceptability, feasibility, adoption, fidelity, implementation cost, penetration and sustainability) we demonstrate feasibility by achieving clinical use across three sites^31^. Acceptability is also demonstrated as reflected in the pathologist survey. Using a ‘before and after’ study design with a staged assistance step to assess AI’s impact on diagnostic decision-making, offers a blueprint for AI deployment in histopathology, echoing similar stepwise approaches in other domains such as mammography^32^, with safety considerations for PaPr deployment detailed in a separate publication^33^.

The diagnostic impact observed (5.4% of authorised reports altered by AI assistance, with 1.3% of changes potentially affecting clinical management) was modest but clinically meaningful. In all, 5 of 386 patients would have received different treatment options when AI assisted in their diagnosis in phase 3 (second read). In 2 cases initially diagnosed as benign, one harboured a GG1 cancer and another was reclassified as ASAP. Despite the AI’s high negative predictive value, it is important that pathologists remain vigilant over time to the possibility of suspicious areas not being predicted as such by PaPr (false negative), as over reliance could result in such ASAP or cancer regions going undetected. One way to mitigate this risk would be for pathologists to continue following standard practice and examine of all the tissue present on the slide. This would be one of an ensemble of responses - including validation and continuous monitoring of system performance, adaptations to pathologists’ training and clinical audit processes - that will need to be adopted if pathologists are to retain agency as decision makers^26,27^. This observation of pathologist behaviour in our study again highlights the importance of understanding the human factors impact when using AI, and the need for this to be understood in order that AI systems can be safely introduced. Such behaviours can then be targeted through education and training, and sharing of experiential learning is essential for safe roll out of AI in clinical practice.

With regards to Gleason Score changes, in 2 patients AS was removed as an option due to an increase in GG, and in one patient it became an option, but the patient opted for radical treatment. Importantly, these changes occurred in the context of specialist NHS centres with expert pathologists. In non-specialist or resource-limited settings, the diagnostic effect of AI may be greater. This is supported by earlier studies showing that AI can improve diagnostic accuracy both in uropathologists and generalists, but more so in the latter^25^. Notably, AI did not override pathologist decision-making but rather highlighted potential areas of diagnostic discrepancy, underscoring the collaborative potential of human-AI interaction. What has become apparent from this study, particularly the GG1 case above, is that pathologists knowing they will be able to view the AI inherently changes the dynamic – that focus had been seen but the pathologist was curious to see the AI output without which they would have requested IHC anyway. Therefore, the concept of AI picking up cancers ‘missed’ by AI or human versus AI is too simplistic to reflect the complexity of pathological decision making which itself is probably altered by having access to the AI. Due to changes in clinical guidelines^34^, GG thresholds (e.g., GG1 vs. GG2) are now less critical for decisions like AS versus radical treatment but there were no GG1 to GG2-5 (or vice versa) reclassifications observed from AI. Also, this study was not designed to assess long-term clinical outcomes, such as metastases or cancer-specific mortality.

The Cohen’s Kappa level of agreement in GG between AI and pathologists was not dissimilar to what might be expected between pathologists. It was 0.5 site A and 0.4 site B and would be higher if GG4 vs GG5 was not considered a difference. Inter-observer Gleason scoring concordance rates in the literature vary in the range of 0.4–0.5 between general pathologists and 0.6–0.7 for GU pathologists^35^, and similar to agreement levels within the study’s own pathologists. In the baseline Gleason Scoring exercise for study pathologists, Fleiss’ Kappa level of agreement was 0.63 (Supplemental Table 8). Prior studies evaluating PaPr have reported greater concordance between Gleason Score in the initial biopsy and the subsequent prostatectomy report when this AI tool was used in the diagnosis of the primary biopsy^36^, but this aspect was beyond the scope of our present work. By the end of the study, 75% of pathologists in the survey suggested confidence in the Gleason Score indicating a learning curve in terms of confidence in grading when doing this in conjunction with AI when compared to the kappa levels of agreement part way through the study. Overall user experience was positive with all stating in the end of study survey that they would use the AI in all or most of their clinical practice, and this does not appear to be impacted by the cohort in terms of prior experience although we acknowledge that this is a self-selected group of pathologists with an interest in AI and were generally quite experienced.

Clear transformational potential has been achieved with this study design, with at least 2 study sites adopting routine AI assisted prostate cancer diagnosis in the long term and the third currently scoping this out.

The decision not to blind the second pathologist in the adjudication process to the reporting pathologist’s opinion on diagnosis and/or Gleason Score was made to make the process as similar to clinical practice as possible, in accordance with this being a prospective real-world evaluation. Pathologists in routine practice would usually ask specific questions on a case, for example on the Gleason Scoring, explaining their options eg a case borderline for 3 + 4/4 + 3 that the pathologist would like a steer on or highlighting a particular focus of interest that they would like an opinion on. This means that a second opinion pathologist is usually not starting blindly ‘from scratch’ in reviewing a case.

Compared to a prior meta-analysis of uropathology AI^9^, which reported high sensitivity and specificity, this study’s AI showed similar sensitivity but more variable specificity, consistent with its training to prioritise cancer detection. However, methodological differences prevent direct comparisons between our work and that meta-analysis, due to this study’s prospective design and focus on real-world clinical performance. Unlike retrospective studies using curated slides, this prospective design used consecutive, unselected cases that aim to capture real clinical diversity (e.g., repeat biopsies, hormonal therapy, slide artefacts). Only very occasional studies have used AI in these more complex settings, e.g., AS^37^. The studies in the meta-analysis also used patch or slide level performance rather than case-level as in our study which better reflects real life practice. Our study reflects true clinical use and human-AI interaction, which retrospective validation studies cannot address. However, to preserve turnaround times, expert consensus panels were not used, limiting the establishment of a single reference diagnosis. Possible explanations for the lower rates of NPV and specificity in phase 3 compared to phase 4 and expected from the literature have been explored in the results section. These include ASAP potentially confounding the NPV data with learning curve and relatively few benign cases affecting the specificity.

It is also noteworthy that this study uses case level interpretation, but case-level comparison against a reference standard is potentially more forgiving than slide- or core-level analysis in terms of sensitivity, but it could be argued less forgiving of specificity where a single false positive within one slide of many indicates a case-level false positive and this effect is reflected in the standalone performance results. The ‘forgiveness’ effect is also impacted by the number of slides and cores per case (more slides, more ‘foregiveness in sensitivity) and we report on average figures where there are site specific differences, as multiple incorrect slide-level assessments may not affect the final case diagnosis.

The most striking findings of this study relate to workflow efficiency. In the fully AI-assisted phase, mean TAT was reduced by 30.1 h at one site (*p* < 0.0001). The site in which these results reached statistical significance showed a mature digital workflow and full IT integration, together with the highest case volume for the study. Site C saw a 29-h reduction in TAT, but this was not statistically significant potentially due to lower caseload in the study. This kind of time saving could be the difference between meeting and not meeting TAT targets and would help alleviate time pressure across NHS trusts. This reduction in TATs is similar to that described previously, whom, in a retrospective evaluation of the same AI tool, showed faster reporting times by 24 h when the pathologists were aided by PaPr^36^. The absence of a reduction in TAT at site B may be explained by their routine double (in-line) reporting process which controls TAT in preparing cases for the MDT. Cases are reported, double reported and authorised in time for the MDT rather than as per the other sites whereby cases are authorised as they are ready without routine double report. It could be argued that at site A, the pathologists had more experience in the end in terms of number of cases seen with AI and duration of time that they had in becoming used to using AI in clinical practice which resulted in a statistically significant reduction in TAT. Articulate Pro involved a learning curve with the AI and an even longer timeframe of deployment and evaluation may see even greater effects.

All 3 sites showed significant reductions in cases requiring immunohistochemistry of between a third and a half of cases (adjusted ORs 0.50,0.43,0.33, *p* < 0.0001, *p* = 0.01, *p* = 0.001) after adjusting for case mix and individual site characteristics which is not typically done in such studies^38^. Reasons for IHC request are complex and usually due to the diagnosis not being certain (59% cases), rather than confirming small areas of cancer^39^. The reduction in IHC requesting is therefore likely due to increased certainty in those cases with AI help. Interestingly, another study that evaluated PaPr also found significant reductions in IHC requests (as well as second opinions) when AI assisted the pathologists^15^. IHC requesting will also be a topic discussed in more detail in the follow on paper on cost effectiveness and needs a further detailed analysis/study to look at the complexity of patterns of requesting in addition to summary statistics.

One of the objectives of Articulate Pro was to assess the transformative potential of this new technology. The impact of AI assistance in diagnosis in this study changed the reporting of 5.4% of cases and 1.3% patients would have been offered a different range of management options as a result. We use the UK as an example, but similar effects on reductions in IHC requesting^38^ and TAT^36^ have been described in studies from other countries (e.g., Netherlands, Portugal respectively). Between 70,000^40^ to 100,000 prostatic biopsies are performed annually in the UK and, if the results of this study were scaled to the entire UK, the use of AI could result in optimised diagnostic reports of 3000–5000 patients every year. Based on our study with the limitation that impact was evaluated at local MDT level and at 2 sites only suggests that across the UK more men would only be offered the most appropriate treatment options. This would help prioritise patients that need radical treatment, reducing unnecessary follow-ups, tests and avoid the morbidity and mortality associated with over and under treatment.

AI usage would eliminate tens of thousands of IHC requests, freeing up valuable lab resources to perform other tasks. Furthermore, the impact of cutting down TATs by one day, could return NHS patients 100,000 days of unnecessary waiting time. Also, our study evaluated the impact of AI assistance in a specialised setting, which is not representative since the majority of prostatic biopsies are diagnosed by non-specialists, which probably underestimates the true populational effect of the new technology. Although we did not assess cost-effectiveness, the reductions in IHC and TAT would imply a strong rationale for deployment, especially when scaled across NHS laboratories already under workforce pressure. Improved efficiency could support national targets for cancer diagnosis and help mitigate delays in patient management caused by service backlogs and pathologist shortages^13^. This, together with the participant pathologists’ strong preference to be assisted by AI as demonstrated by the survey taken, further reinforces the feasibility of using this technology as a viable diagnostic aid.

Several limitations must be acknowledged. First, we did not assess downstream patient outcomes or conduct a formal health economic analysis, which correspond to the upper levels (5 and 6) of the AI efficacy framework^29^ and remain essential areas for future work with a dedicated paper to follow on cost effectiveness (level 6). Second, although all sites operated in digital workflows, there were differences in digital maturity and infrastructure, which may have influenced the extent of AI benefit, particularly regarding TAT. Third, technical performance issues (e.g., slide digitisation failures or AI downtime) were not systematically recorded, although overall AI availability was high. Fourth, we did not include a consensus reference diagnosis due to the practical constraints of real-time reporting and laboratory turnaround standards. Finally, although the AI system evaluated was FDA cleared, CE-marked and externally validated^22,23,25^ its commercial nature introduces a potential source of bias^41^. Nevertheless, academic independence was maintained throughout. While Paige was involved in study design and manuscript elaboration, it was not involved in data analysis and interpretation to avoid bias.

To ensure the technical integrity of the study, where technical issues were identified, they were mitigated. Although we focussed on AI platform specific issues, the AI is inherently reliant on a robust DP workflow and it is beyond the scope of this paper to discuss that in detail. Issues included image-level artefacts (e.g., WSI artefacts like blurring, or file corruption), interoperability constraints between the AI inference engine and the DP infrastructure, and network infrastructure instabilities. Additionally, administrative hurdles such as user authentication failures and software configuration errors were monitored. Site A with full integration needed to ensure optimal exporting procedure with robustness of the interface including image and metadata export. Prompt exporting was key to ensuring results were available when reporting, in other words, a timely and robust result feed. Despite these potential risks, the platform maintained very high availability throughout the study period, with only nominal disruptions related to routine network routing and credential management. A summary of potential obstacles and their mitigating actions is shown in Supplemental Table 9.

In conclusion, this study provides robust evidence that subject to appropriate steps being taken in pathologist training, system monitoring and clinical audit, prospective diagnostic AI can be safely and effectively implemented into NHS histopathology workflows. In addition, it provides a blueprint to assess the impact of AI use in clinical practice and adds pertinent aspects of human factors to supplement the limited existing literature and white papers^42,43^. By improving efficiency, reducing IHC testing, and enhancing diagnostic accuracy, this AI tool has the potential to facilitate faster, more precise reporting at scale. If broadly implemented across prostate cancer diagnostics in the NHS, such technology could deliver tangible system-wide benefits, freeing up laboratory capacity and optimising treatment selection while shortening waiting times for patients.

## Methods

### Datasets for analysis

This observational service evaluation study was conducted at 3 National Health Service (NHS) hospitals in the UK; Oxford University Hospitals NHS Foundation Trust; University Hospitals Coventry and Warwickshire NHS Trust; and North Bristol NHS Trust which have been redesignated in the paper as sites A, B and C in a random order to preserve site anonymity. All sites are academic teaching hospitals and have validated primary diagnosis using DP for prostate biopsy reporting.

### Ethics and consent

External ethical review and consent were not required as the study design was a (post market) service evaluation of AI as part of SoC in the diagnostic pathway. The AI was only used with the oversight of a pathologist and not autonomously. All governance aspects were ratified by the lead site. Data analysis was conducted using de-identified data. Although not a randomised controlled trial, the study was registered under International Standard Randomized Controlled Trial Number (ISRCTN) 91685765. Patients are not identifiable from the material. The research was performed in accordance with the Declaration of Helsinki.

### Inclusion and exclusion criteria

The study cohort included all patients undergoing a prostate biopsy (including local anaesethic transperineal (LATP) and transrectal ultrasound-guided (TRUS) biopsy), at each of the 3 hospitals. Cases were consecutive as far as was practical within the logistical constraints of a clinical workflow. Cases included the index (first) biopsy or any follow up biopsy. Case exclusion criteria included temporary technical issues preventing use of software systems, and cases with WSIs that did not meet image quality requirements which could not be resolved by rescanning. Sample size was determined by feasibility. Pathologists recorded technical system errors, excluded cases, and recorded reasons for their exclusion.

The analysis of data was done at the case (patient) level ie the overall interpretation of the case rather than slide level (the diagnosis on each slide in a case) as the former more closely replicates real life practice.

### Participating pathologists

All pathologists in the study were certified specialist urological pathologists with varying degrees of exposure to uropathology and DP. At site C there was limited exposure to previous AI assisted pathology reporting. To ensure standardisation of pathology reporting across sites and pathologists, a set of criteria was implemented for study pathologists (Supplementary Table 8).

### AI system

The Paige Prostate (PaPr) AI system is composed of three different modules that operate in synchrony. This study investigated the modules PaPr Detect (v1.2.0) and PaPr Grade and Quantify (v2.2.0). A third module, PaPr PNI, is concerned with the detection of perineural invasion (PNI). These modules are based on a deterministic deep learning approach, trained with digitised H&E prostate needle biopsy slides diagnosed at Memorial Sloan Kettering Cancer Center in New York, USA. Its development and performance have been described previously^21,22,25^. Briefly, PaPr was trained using a weak label approach called multiple instance learning, where the digitised WSIs were matched with their corresponding pathology report, which acted as weak labels for each slide. PaPr processes WSIs both during training and inference at a resolution of 0.50 microns per pixel. If the slides are acquired at 0.25 microns per pixel, the system downsamples them to 0.50 microns per pixel for the inference pipeline.

PaPr Detect outputs a binary classification of suspicious or not suspicious for harbouring cancer for each WSI, and if a WSI is suspicious, it displays a single focus location with the greatest statistical evidence for suspicion of cancer, marked with a crosshair (Supplementary Fig. 3a). PaPr Detect is not trained to separately classify atypical small acinar proliferation (ASAP)^23,25^. ASAP is a category that can be assigned to small areas in prostate biopsies that are suspicious, but not conclusively diagnostic of adenocarcinoma. PaPr Detect is FDA-approved (DEN200080) for in vitro diagnostic use in the USA as a second-read device, to confirm the pathologist’s adenocarcinoma diagnosis. In the UK and Europe, PaPr has UKCA and CE-IVD marks for the scanner image formats used in this study.

PaPr Grade and Quantify calculates tumour percentage, tumour length, and Gleason percentage. Furthermore, it can ghost out benign tiles thus highlighting suspicious areas, which directs the reviewer’s attention to the regions harbouring suspicious tissue (Supplemental Fig. 3b). The system includes tissue overlays showing predicted locations for each Gleason pattern for the pathologist to review. PaPr has not been trained to discriminate between cribriform patterns from other Gleason 4 patterns, and thus cribriform areas are predicted as Gleason 4. Similarly, intraductal carcinoma is predicted as suspicious tissue, but no specific label is shown when it is present.

The third module is called PaPr PNI and detects regions suspicious for perineural invasion (PNI) on a WSI level. It operates in a similar fashion to PaPr Grade and Quantify, and when PNI is predicted, it displays a message warning and can ghost out regions with no PNI to direct the viewer’s attention to the regions where PNI is suspected (Supplementary Fig. 3c).

The outputs of PaPr are displayed in the FDA-approved Paige FullFocus web-based viewer system. No site-specific tuning nor calibration of the algorithm was undertaken, and the same system was used across the sites without modifications to ensure consistency.

### Study sites

2 sites used the Philips IntelliSite DP platform for slide scanning (Ultrafast Scanners) and primary H&E viewing. One of these sites exported WSIs of study cases overnight using an export tool with daily frequency and accessed the AI modules and results on study cases from a worklist within FullFocus, and another site launched the AI system directly from the local LIMS in real time and served as a demonstration of full integration for clinical workflows. The third site scanned WSIs with the Hamamatsu S360 scanner and used the Paige FullFocus viewer both for primary reporting and accessing AI modules.

Sites A, B and C are all academic (teaching) centres operating sub-specialist uropathology reporting. Site A achieved full workflow integration with the LIMS as above, which was facilitated by having a Filemaker Pro system maintained by a local IT team. The total case volumes per lab per year in the period 2022–2024 were: site A 60,000, site B 70,000, site C 30,000 (approximately). Prostate biopsy volumes were site A 1000, site B 1200, site C 400 cases approximately per year. Site B has an ‘in-line’ MDT review process which controls TAT by this routine double reporting process. The number of core biopsies per case separated by site is shown in Supplemental Table 10 showing no statistical significant differences between phases 1 and 4 in mean number of cores per case at any of the sites.

### Endpoints (outcomes)

The primary endpoint was to determine the impact of PaPr in diagnostic assistance capturing impact on final authorised reports, clinical management decisions and pathologist experience.

The secondary endpoints were to determine impact on workflow and resource utilisation due to PaPr assistance and evaluate standalone performance (cancer/ASAP versus benign/PIN and Gleason Score).

### Study phases (Fig. 1)

The 4 study phases were a stepwise deployment and evaluation of the AI in the workflow:

Phase 1: Baseline, no AI

Phase 2: Second-read pilot (small scale pilot conducted before phase 3 to familiarise pathologists with the AI and SCT

Phase 3: Second-read full study (staged introduction of AI)

Phase 4: Concurrent-read (full AI-assisted review/on demand use)

The study design progressively introduced AI assistance into prostate biopsy reporting. In Phase 1 (baseline, no AI), SoC practice was characterised in terms of workflow steps, resource use, time required and final diagnosis, to establish a baseline comparison for Phase 4 (concurrent-read/full AI assistance).

Phase 2 (second-read pilot) was a small-scale pilot to familiarise the pathologists with the AI, the SCT and processes for phase 3 (second-read full study). This data was captured and phase 2 cases were counted in the total number of cases reported with AI, however, other data was not analysed in the statistical analysis as there was too little to be meaningful. In this phase any issues that arose were addressed and thus was not representative of the true diagnostic process or workflow.

Phase 3 (second-read full study) used staged assistance with AI and the emphasis was on capturing impact on diagnosis rather than workflow factors. Pathologists first diagnosed the case without AI including any extra work such as IHC, deeper levels (DL) or further opinions that might be required. After locking down a report in the SCT but before formally authorising the case, pathologists accessed the AI outputs. The outputs included binary classification of WSIs as either suspicious or not suspicious with overlays on the WSIs indicating areas of suspicion, or suspicious WSIs, a Gleason Score and overlays highlighting Gleason patterns. Pathologist-AI discrepancies in case level diagnostic classification and case level maximum Grade Group (GG) according to protocol definitions were recorded and adjudicated, prior to final case authorisation.

GG4 versus GG5 was not considered a discrepancy needing adjudication because this distinction is usually of limited impact on treatment decision making. Although prognostically it could be of importance, the focus in this study was on immediate clinical management decisions. The contemporary nature of the study without the 5–10 years of follow up needed to establish impact on clinical outcomes in prostate cancer and thus not being an endpoint, was the justification for this approach. Adjudication could be IHC or further pathologist opinion depending on which was felt to be most appropriate by the reporting pathologist. The further opinion pathologist was not blinded to the output of the reporting pathologist or PaPr. Pathologists also had the option to choose to adjudicate cases upon viewing the AI outputs when adjudication was not necessitated by the protocol.

The definition of a case level diagnostic discrepancy requiring adjudication was when a suspicious flag from Paige was the only flag on a case and if proven correct would change the case from benign/PIN to ASAP/cancer (or vice versa when no suspicious flag but pathologist opinion was ASAP/cancer). The pathologist was able to judge whether to disregard flags on other specimens/cores if they did not agree, on cases that were already clearly malignant in a different part of the case.

The definition of a case level grading discrepancy was between the maximum/highest Gleason Score/GG offered by Paige and the pathologist pre-Paige ‘bottom line’ maximum/highest Gleason Score. Gleason discrepancies were only considered in areas which were definitely seen as malignant by the pathologist, i.e., if a higher Gleason was offered by Paige on an area that was considered benign/PIN/ASAP, this was disregarded.

Phase 4 (concurrent-read) involved fully AI-assisted reporting, pathologists were able to access AI outputs as desired throughout case review (on-demand use) and had visibility from the outset.

To interpret the possible case-level impact of AI to clinical management, sites convened a Discrepancy Board for phase 3 with composition representing the MDT setting: including pathologist, urologist and/or oncologist.

### Pathologist training and validation

Prior to using the AI in the study, all 11 participating pathologists received training on the software, SCT and PaPr. Basic proficiency and system familiarity was acquired through mandatory pathologist-led software training sessions, provision of training documentation, access to a software testing environment and example WSIs and cases. Pathologists viewed approximately 20 cases in a training set selected to be representative of good or difficult examples of diagnoses, grade or AI outputs (site A examples of training set in Supplementary Table 11). Pathologists then undertook phases 2–4 of the study. This framework could serve as a mechanism for AI validation by pathologists following the principles of reflective learning to determine length of staged assistance.

### Data capture and validation

A customised electronic data capture system (SCT) was designed and built for the study using the Grails open-source web application framework and the PostgreSQL. The Spring Security framework was used to provide both authentication and authorisation. In addition, audit functions were used to trace user activity on the SCT. The SCT was hosted on a site A NHS virtual server. An SSL certificate was installed on the server that authenticates the SCT identity and enables an encrypted connection. SCT was accessible on the Health and Social Care Network (HSCN) to the other centres. For all study phases, key metrics describing the case information, time/date stamps and patient characteristics were recorded in the SCT (Table 1) in de-identified form. All study phases also collected data related to the final authorised report.

### Data analysis

Summary descriptive statistics are presented with number of cases, mean and standard deviation (s.d.) for normally distributed continuous variables and number of cases/total (percentage) for counts. Pearson’s Chi squared test was used to compare the distribution of cases into the three diagnostic categories and across the GG categories in Phase 1 with Phase 4 within each site. The measure of agreement between PaPr AI assessment and unassisted assessment by the pathologist for the maximum GG was the Cohen kappa statistic. The TAT in the diagnostic process in phase 1 was compared with phase 4 within sites using a regression analysis of time with diagnostic category and phase the independent variables. The results of the t-test for the comparison of phase 4 with phase 1 are reported. The proportions of cases requiring IHC between phases within sites were compared using logistic regression with diagnostic category and phase the independent variables.

The standalone performance of AI at the case-level in the classification of prostate cancer was assessed using sensitivity, specificity, negative predictive value (NPV) and positive predictive value (PPV) for phases 3 and 4 with the final authorised diagnosis as the reference standard. The standalone AI performance calculations have the limitation that the reference standard (final authorised diagnosis ie pathologist plus AI) was not adjudicated in all cases – in phase 3 only discrepant cases and in phase 4 no adjudication was mandated.

The following definitions were used:

True positive. Reference diagnosis is positive (adenocarcinoma/ASAP) and there are one or more suspicious AI area(s) in the case

True negative. Reference diagnosis is negative (benign/PIN) and there are no suspicious AI areas in the case

False positive. Reference diagnosis is negative (benign/PIN) and there are one or more suspicious AI areas in the case

False negative. Reference diagnosis is positive (adenocarcinoma/ASAP) and there are no suspicious AI areas in the case

The agreement between the standalone outcomes from AI and the pathologist assessment of maximum GG at case level was assessed using a Cohen’s Kappa.

### Patient involvement

We involved patients and the public who have experience of prostate cancer at all stages of the study. Patient views were used to help in the writing and designing of the project application including input into governance processes, and 3 prostate cancer patients/survivors participated in the study as members of the Project Management Board. Our patient representatives made significant contributions to the study and have been involved in key decision-making processes throughout. We consider their views to be important to the work of the study and crucial to ensuring the study remained patient-centric.

### Human factors

All study pathologists were invited to complete surveys at baseline (pre-study), during the study and an end of study survey which included questions related to acceptability of the use of AI. Pathologists at site A were also interviewed and observed using previously described methods^26,27^. The results of the survey, interviews and observational work will be presented in more detail in further dedicated manuscripts. Pertinent survey findings are presented here in brief to supplement the main study findings.

The survey questions were developed by LB and CV, with input from the study team. The questions were largely of Likert scale-type with response options on a continuum from “Strongly disagree” to “Strongly Agree”, together with a “ranking” question related to the subjective utility of the AI. There was opportunity for free text responses. Pathologist surveys were distributed via an email link to the online SurveyMonkey® survey tool (www.surveymonkey.com).

Other clinician and patient views have been ascertained in a separate survey study and have been published separately^28^.

## Supplementary information

Supplementary Information

## Data Availability

The datasets generated during and/or analysed during the current study are not publicly available due to reasons of sensitivity and confidentiality. De-identified study data could be made available via the corresponding author on reasonable request. Data is located on secure computing storage at Oxford University Hospitals NHS Foundation Trust and University of Oxford.

## Code availability

Paige Prostate Detect and Paige Prostate Grading and Quantifying are proprietary artificial intelligence software applications that are commercially available as Software as a Medical Device (SaMD) under the UK MHRA. Accordingly, no custom computer code was used to generate the results reported herein. The underlying code of the SaMD applications used in this study (i.e., Paige Prostate Detect and Paige Prostate Grading and Quantifying) and their respective training datasets are not publicly available for proprietary reasons.
