# Supplementary material for: An evaluation of artificial intelligence assisted prostate biopsy reporting in the Articulate Pro study
Source: NPJ Digit Med. 2026 May 22;9:537. doi: 10.1038/s41746-026-02592-8 (PMC13365413; doi:10.1038/s41746-026-02592-8)
Supplement: Supplementary file 1 — Supplementary Information [file 41746_2026_2592_MOESM1_ESM.pdf]

Supplemental Material

Supplemental Table 1

	Benign	Cancer	Atypia	Other	Total
Site A	137 (21%)	486 (76%)	21 (2%)	0 (0%)	644
Site B	279 (24%)	848 (71%)	62 (5%)	0 (0%)	1189
Site C	248 (38%)	361 (55%)	5 (1%)	38 (6%)	652
Total	664 (27%)	1695 (68%)	88 (4%)	38 (1%)	2485

Supplemental Table 1

Pre-baseline audit figures for sites during 2021 (pre-phase 1). In order to evaluate the reporting practices in terms of diagnostic category between the 3 sites, prior to starting the Articulate Pro study, an audit was undertaken during the year 2021. Data on Gleason Scoring was also collected, but only available for site A and thus not shown. Rates of malignancy reporting varied from 55-76% across sites.

Supplemental Table 2

SITE	PHASE	TOTAL NO. OF CASES	NO. OF CASES EXCLUDED	FINAL NO. OF CASES
A	1	316	13	303
A	2	25	0	25
A	3	262	12	250
A	4	342	6	336
SITE A: Total Number of cases in study / total excluded		945	31	914
Excluded cases in site A were broken down into the following categories:<br>Phase 1: Testing = 5; Data entry = 2; Other = 6<br>Phase 3: Testing = 2; Data entry = 4; AI Platform related = 5; Other = 1<br>Phase 4: Data entry = 2; AI Platform related = 4				
B	1	137	1	136
B	2	13	0	13
B	3	137	1	136
B	4	159	2	157
SITE B: Total Number of cases in study/ total excluded		446	4	442
Excluded cases in site B were broken down into the following categories:<br>Phase 1: Data entry = 1<br>Phase 3: Data entry = 1<br>Phase 4: Data entry = 1; AI Platform related = 1				
C	1	131	6	125
C	2	22	1	21
C	3	N/A	N/A	N/A
C	4	116	5	111
SITE C: Total Number of cases in study/ total excluded		269	12	257
Excluded cases in site C were broken down into the following categories:<br>Phase 1: Data entry = 6<br>Phase 2: Data entry = 1<br>Phase 4: Data entry = 1; AI Platform related = 4				
**TOTALS**		**1660**	**47**	**1613**

Supplemental Table 2

Breakdown of included and excluded cases per site and by phase. 47 cases were recorded as being completely excluded (registered as excluded in study capture tool (SCT)) due to a fundamental issue with including the case in the study, for example due to a duplicate case entry or a technical issue. 14 of the 47 cases were for AI-platform related reasons (AI not available, bridge not available or slides/cases not uploaded or missing).

Supplemental Tables 3a-3c

Supplemental Table 3a

SITE A	Diagnostic category		
	Phase 1	Phase 4	Total
Benign/PIN	54	66	120
ASAP	18	13	31
Adenocarcinoma	231	257	488
Total	303	336	639
Pearson's chi2(2) =1.69 p=0.43<br>Significant association between phase 1 and phase 4			

Supplemental Table 3b

SITE B	Diagnostic category		
	Phase 1	Phase 4	Total
Benign/PIN	35	34	69
ASAP	4	5	9
Adenocarcinoma	97	118	215
Total	136	157	293
Pearson's chi2(2) =0.68 p=0.71<br>Significant association between phase 1 and phase 4			

Supplemental Table 3c

SITE C	Diagnostic category		
	Phase 1	Phase 4	Total
Benign/PIN	43	26	69
ASAP	6	9	15
Adenocarcinoma	76	76	154
Total	125	111	236
Pearson's chi2(2) =3.97 p=0.14<br>Significant association between phase 1 and phase 4			

Supplemental Tables 3a-c

Comparison of diagnostic categories (cancer, ASAP or benign/PIN) between phase 4 (concurrent-read/full AI assistance) and phase 1 (baseline/no AI). Site A shown in 3a, site B in 3b and site C in 3c. There was significant association between phases 1 and 4 at sites A,B and C representing no major diagnostic shifts with AI deployment (p=0.43, p=0.71, p=0.14 respectively).

Supplemental Figure 1

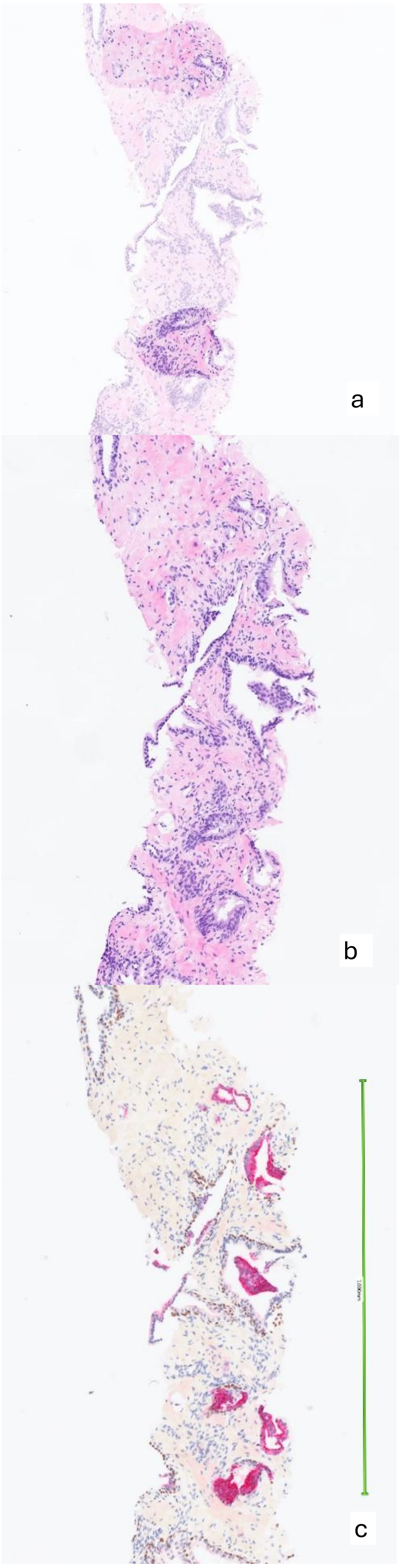

Supplemental Figure 1

A change to the final authorised diagnosis as a result of second-read with AI. A phase 3 case initially diagnosed by the pathologist as benign and upon viewing AI outputs after a suspicious area was identified by the AI, the case was changed to Atypical Small Acinar Proliferation (ASAP). Green scale bar is 1 mm. H&E shown together with output of AI with suspicious area highlighted (1a) and a P63/AMACR ‘cocktail’ stain (1c).

Supplemental Tables 4a-4c

Supplemental Table 4a

SITE A	Grade Group		
	Phase 1	Phase 4	Total
1	23	27	50
2	110	135	245
3	54	36	90
4	17	23	40
5	26	35	61
total	230*	256	487
Pearson's chi2(4) =7.39 p=0.12<br>Significant association between phase 1 and phase 4			

*1 case not included as on hormone therapy, thus GG not appropriate

Supplemental Table 4b

SITE B	Grade Group		
	Phase 1	Phase 4	Total
1	29	32	61
2	40	54	94
3	16	19	35
4	2	4	6
5	7	7	14
total	94	116	210
Pearson's chi2(4) =0.86 p=0.9<br>Significant association between phase 1 and phase 4			

Supplemental Table 4c

SITE C	Grade Group		
	Phase 1	Phase 4	Total
1	10	8	18
2	31	32	63
3	14	9	23
4	3	8	11
5	16	17	33
total	74	74	148
Pearson's chi2(4) =3.63 p=0.46<br>Significant association between phase 1 and phase 4			

Supplemental Tables 4a-c

Comparison of Grade Groups (GG)s between phase 4 after full AI deployment with a concurrent-read and phase 1 (baseline, no AI). Site A shown in 4a, site B in 4b and site C in 4c. There was significant association between phases and thus no statistically significant difference between the phases in the pattern of GG assignment at sites A,B,C (p=0.12, p=0.9, p=0.46 respectively).

Supplemental Tables 5a & 5b

Supplemental Table 5a

SITE A PHASE 3						
Maximum grade group	Unassisted					
Paige	1	2	3	4	5	total
1	28	16	2	0	0	46
2	0	62	12	1	0	75
3	0	0	4	0	1	5
4	0	5	18	12	6	41
5	0	1	3	3	11	18
Total	28	84	39	16	18	183

Supplemental Table 5b

SITE B PHASE 3						
Maximum grade group	Unassisted					
Paige	1	2	3	4	5	total
1	13	3	1	0	0	17
2	2	22	6	0	0	30
3	0	4	2	0	0	6
4	1	5	11	7	0	24
5	0	0	6	9	6	21
Total	17	34	25	16	6	98

Supplemental Tables 5a & 5b.

Case level unassisted pathologist Grade Group (GG) together with the standalone Paige Prostate (PaPr) GG for sites A (5a) and B (5b) in phase 3 (second-read/staged assistance with AI) (data not available for site C as no phase 3 data). In site A, there were 183 cases with pathologist and a Paige GG, agreement for 117/183 (Kappa 0.51 (SE 0.04), agreement percentage 63.2%). 2 cases could not be analysed as there was no GG due to hormonal therapy. In site B, there were 98 cases with pathologist and a Paige GG, agreement for 50/98. 3 cases could not be analysed, one because of hormonal therapy, agreement percentage 51.0%, kappa 0.38 (SE 0.05)

Supplemental Figures 2a-2l

Supplemental Figure 2a. Site A

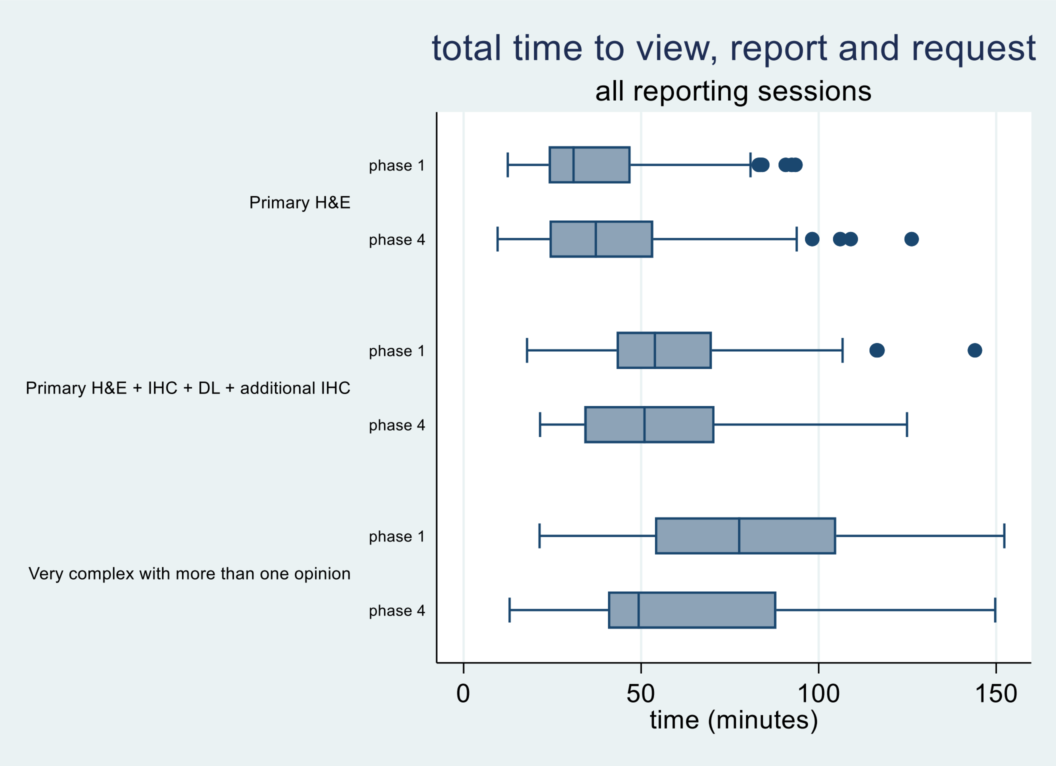

Supplemental Figure 2b. Site A

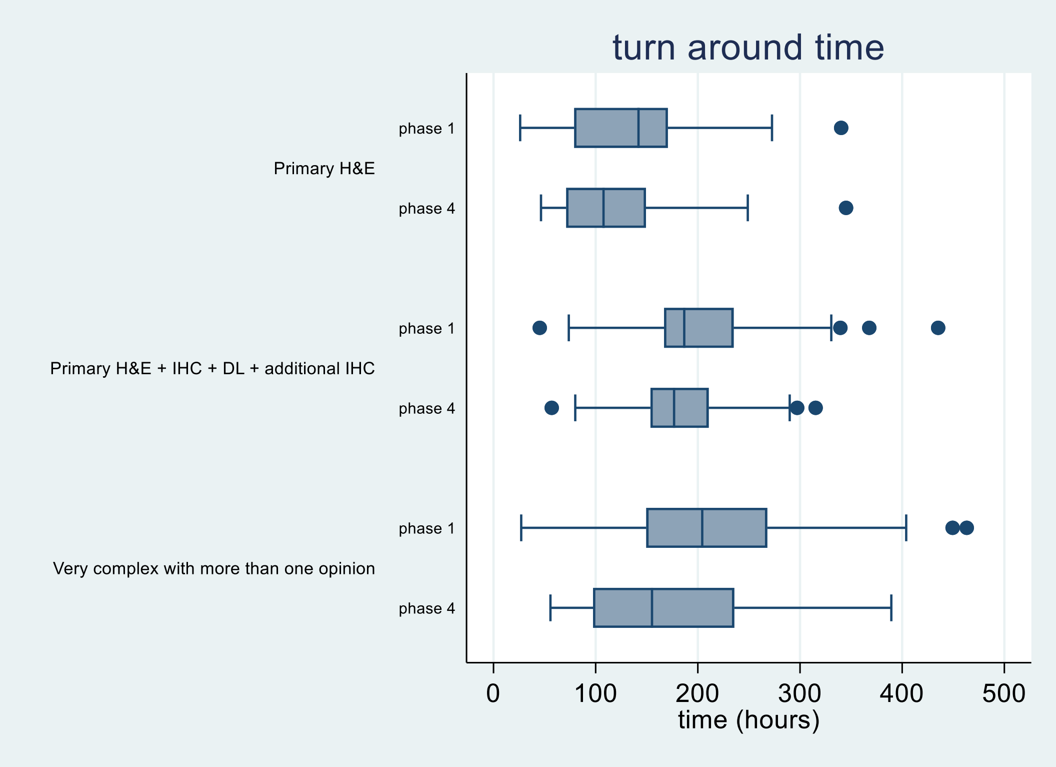

Supplemental Figure 2c. Site A

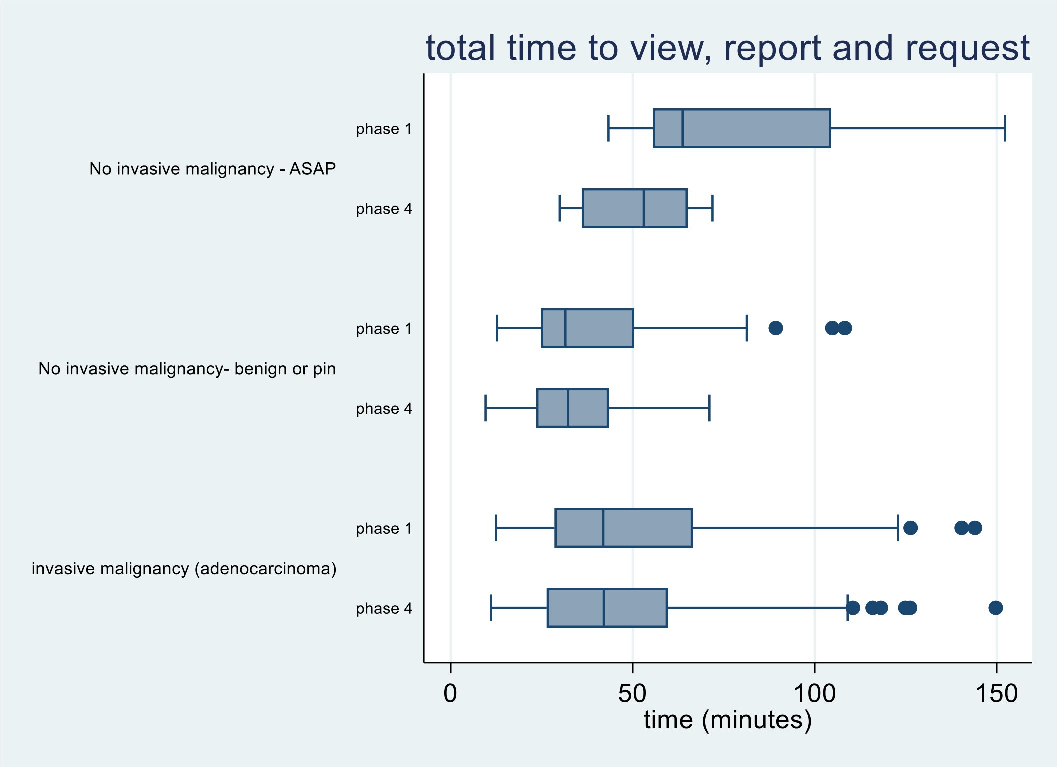

Supplemental Figure 2d. Site A

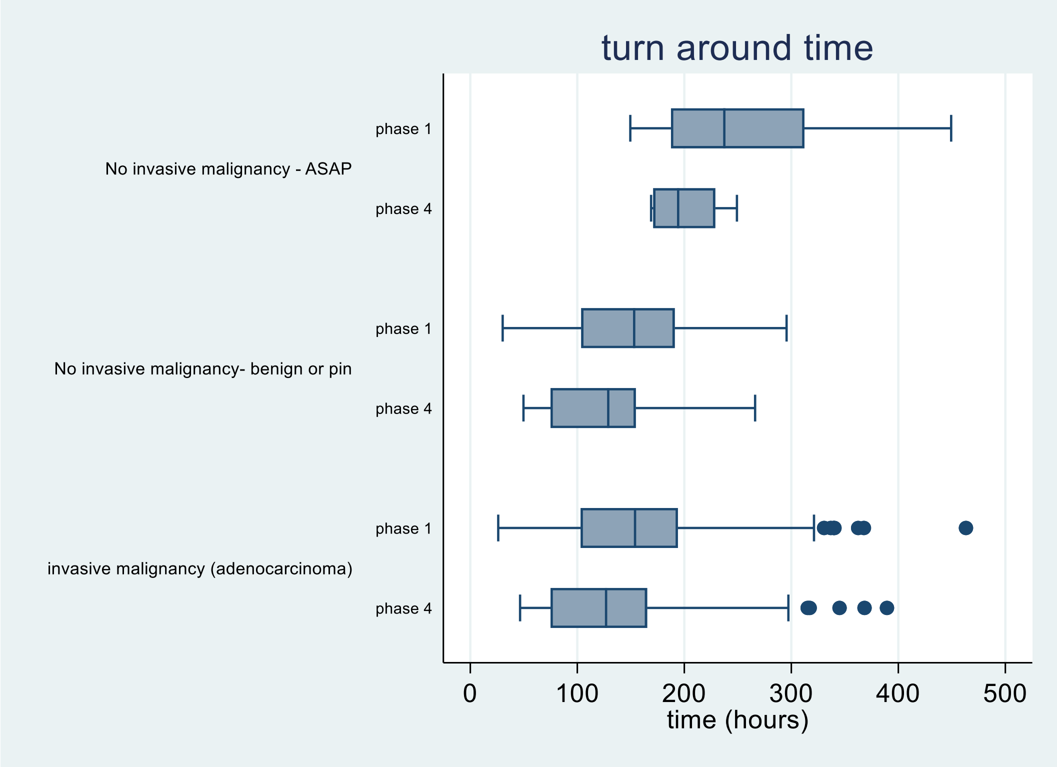

Supplemental Figure 2e. Site B

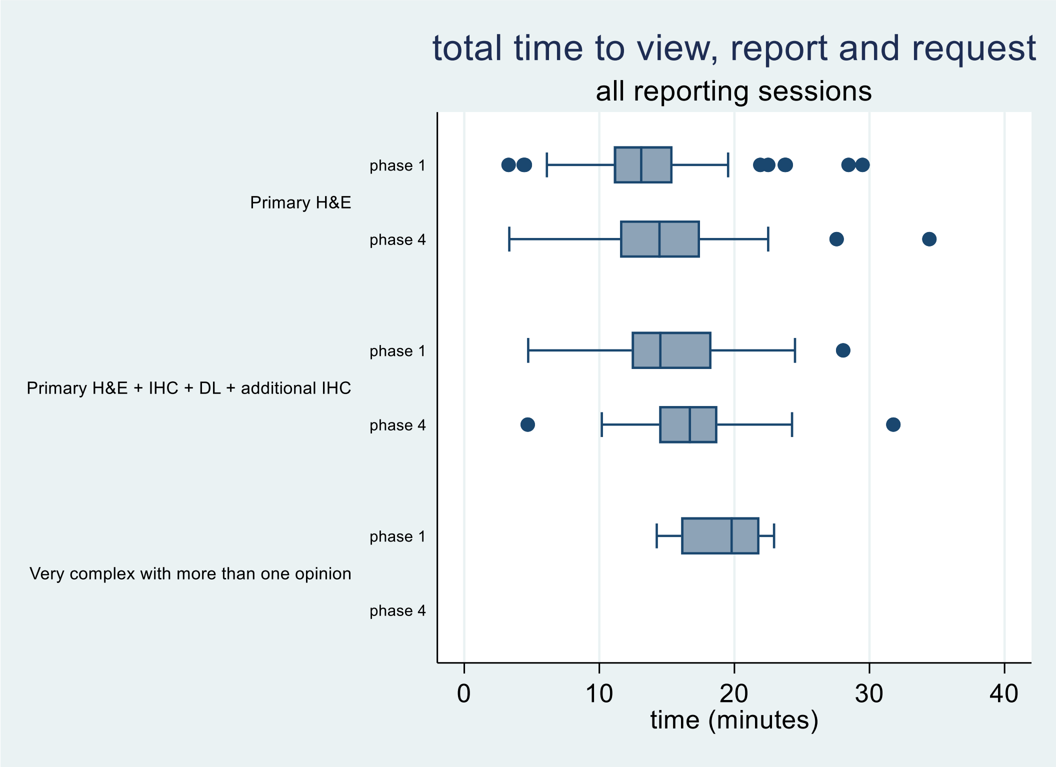

Supplemental Figure 2f. Site B

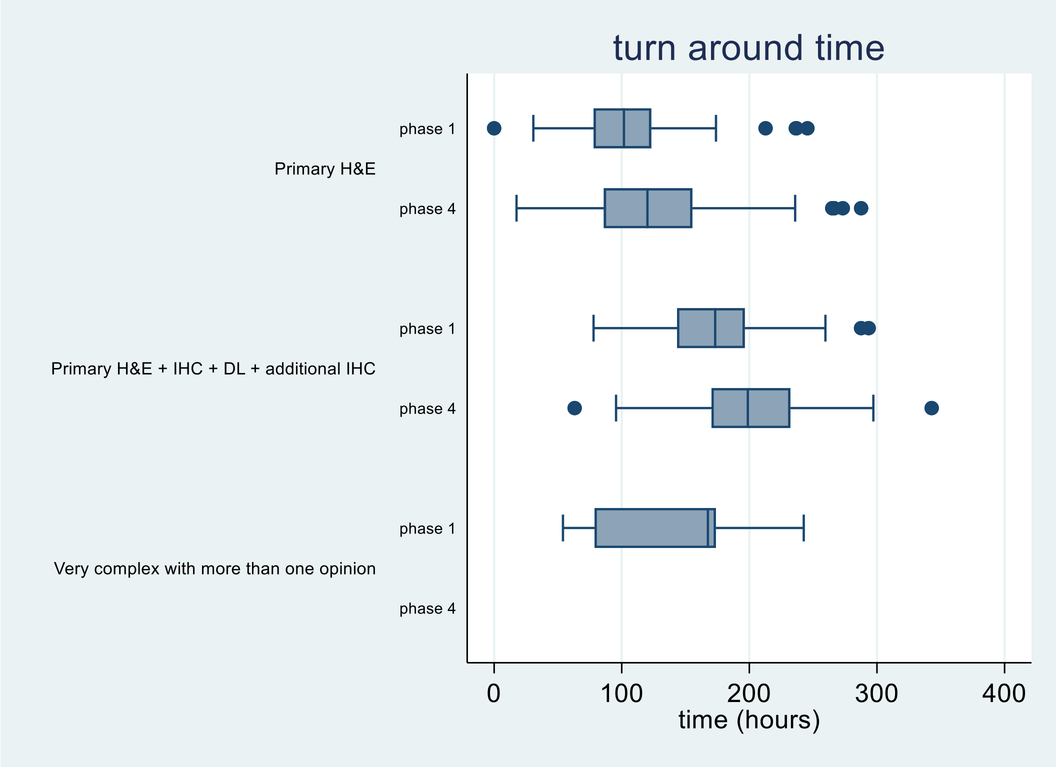

Supplemental Figure 2g. Site B

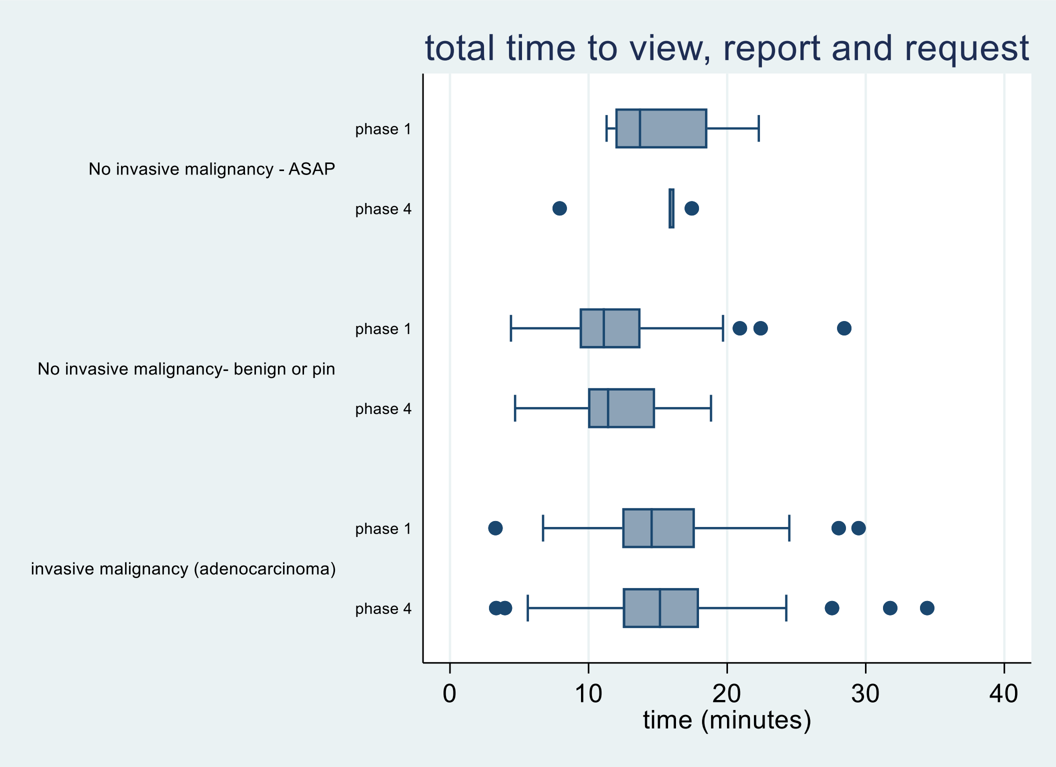

Supplemental Figure 2h. Site B

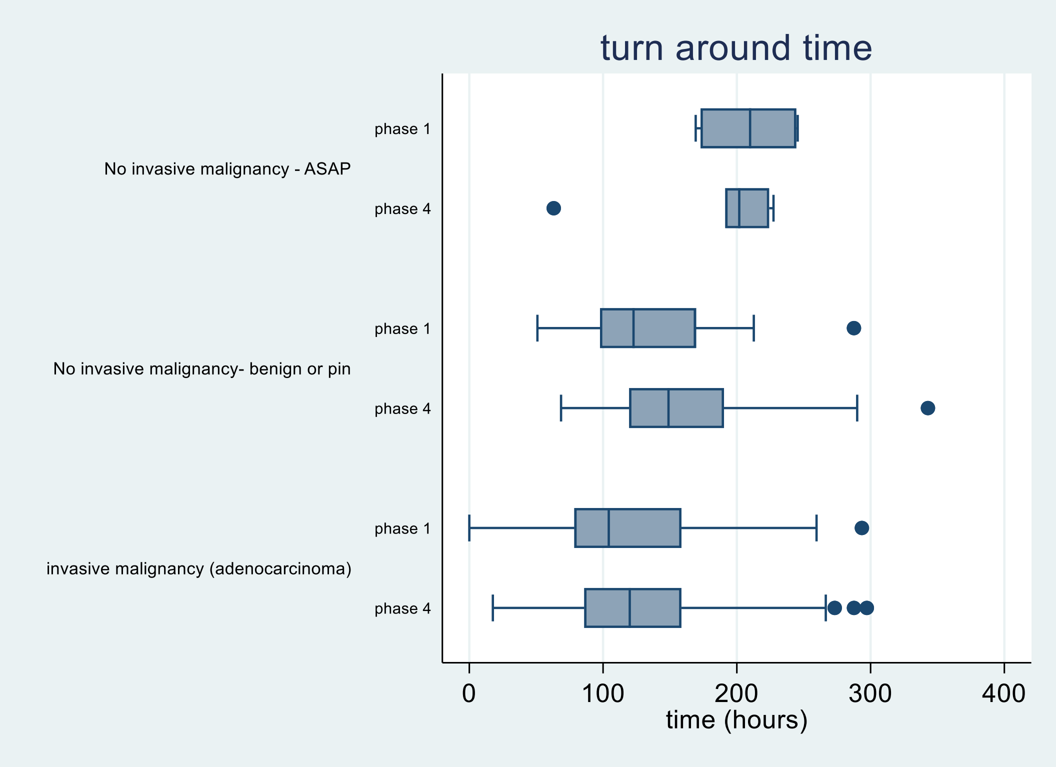

Supplemental Figure 2i. Site C

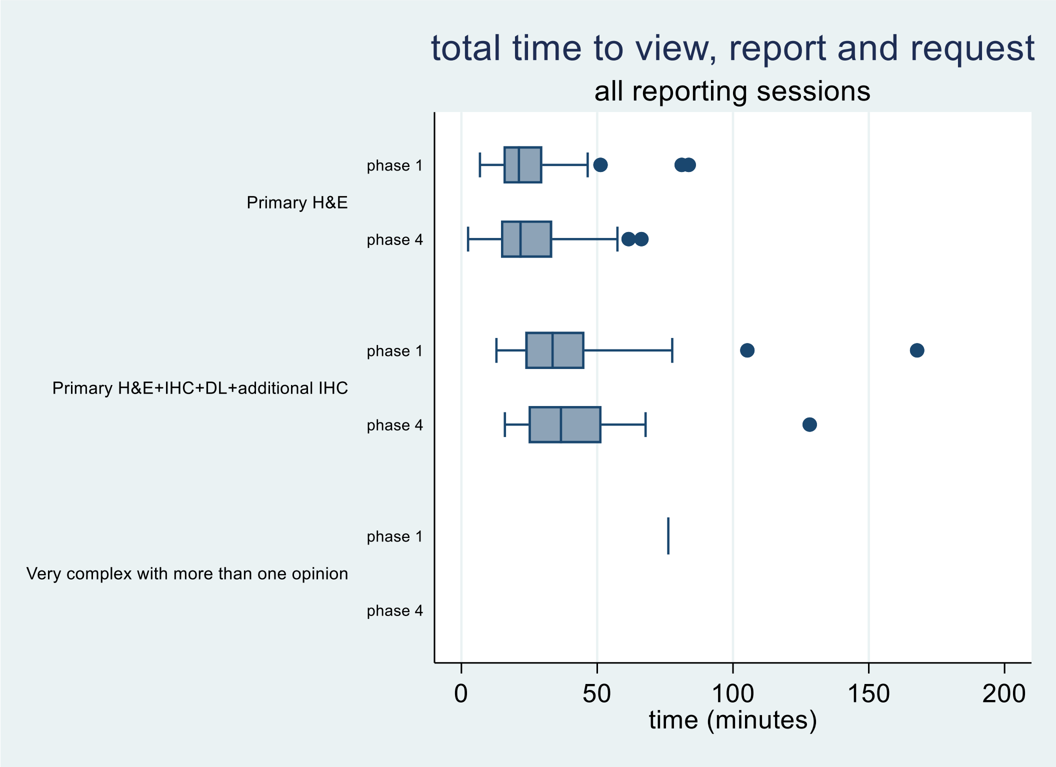

Supplemental Figure 2j. Site C.

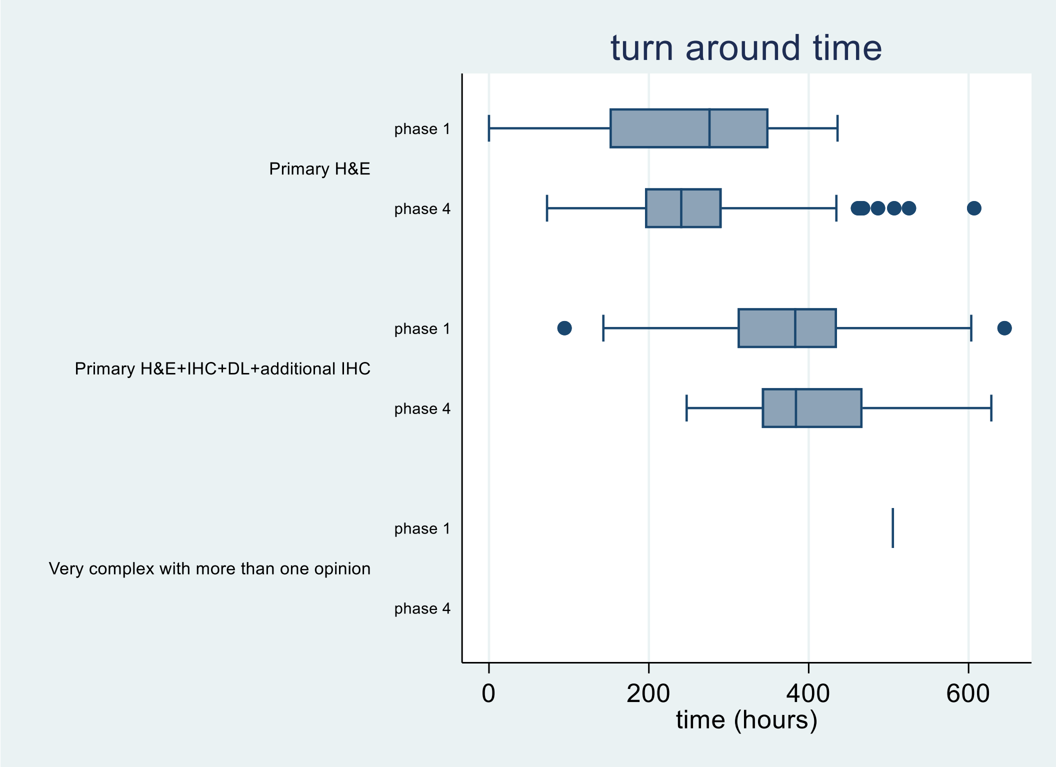

Supplemental Figure 2k. Site C

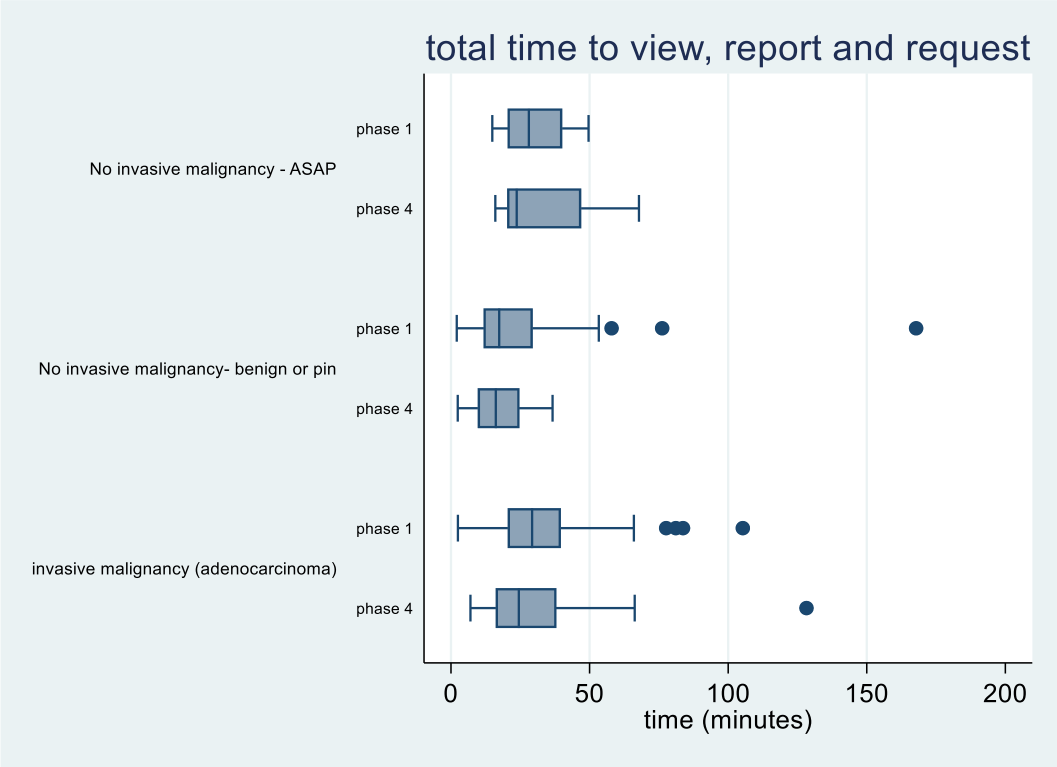

Supplemental Figure 2l. Site C

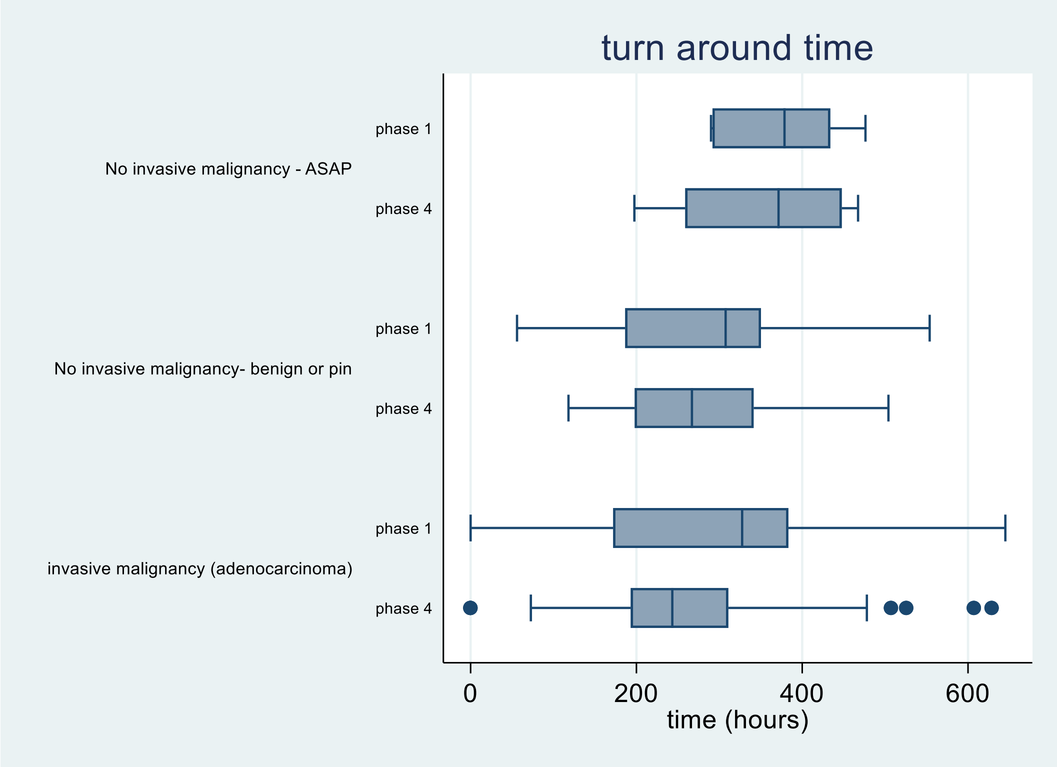

Supplemental Figures 2a–2l

Detailed breakdown by box plot of phase 1 (baseline/no AI) versus phase 4 (full AI assistance with concurrent-read) of the total time to view, report (type, dictate or enter report) and request and also by turnaround time per site and per case shown by the complexity of the case as either reported on H&E only (primary H&E), or that immunohistochemistry (IHC) and/or deeper levels (DL) were requested, or that the case complexity required a further pathologist opinion and also shown by case diagnosis. One outlier case with a reporting time of over 16 hours was excluded from site C as this was not a plausible reporting time.

Supplemental Table 6

Table 6. Turnaround time (hours)				
	Phase 1		Phase 4	
Pathologist	Number	Mean TAT (sd)	Number	Mean TAT (sd)
Site A Pathologist 1	78	139.8 (89.7)	44	113.7 (63.6)
Site A Pathologist 2	118	201.4 (72.5)	111	148.2 (63.5)
Site A Pathologist 3	50	150.1 (65.1)	122	130.4 (49.6)
Site A Pathologist 4	57	130.6 (60.8)	59	110.8 (71.1)
Site B Pathologist 1	61	136.9 (70.8)	60	136.9 (50.1)
Site B Pathologist 2	27	105.9 (37.4)	59	121.8 (39.0)
Site B Pathologist 3	48	142.1 (56.1)	38	166.7 (81.0)
Site C Pathologist 1	29	290.7 (96.9)	21	285.6 (117.8)
Site C Pathologist 2	17	242.9 (135.4)	29	308.7 (121.5)
Site C Pathologist 3	78	312.4 (144.2)		
Site C Pathologist 4			36	237.3 (73.6)
Site C Pathologist 5			25	273.3 (143.9)

Supplemental Table 6

A breakdown of turnaround time (TAT) by pathologist at each of the 3 sites. Note that in sites A and B the study pathologists were the same for phases 1 (baseline/no AI) and 4 (full AI assistance with concurrent-read), but in site C, only 2 pathologists participated in both phases 1 and 4 to enable a comparison of those phases. In site A which showed a statistically significant reduction in overall mean TAT, all 4 pathologists also showed a reduction in TAT. In site C, where a reduction was seen that was not statistically significant, a reduction is seen for pathologist 1, but not pathologist 2.

Supplemental Table 7

	Number of cases	Total number IHC	Mean number IHC/case
Site A phase 1	303	461	1.52
Site A phase 4	336	238	0.71
Site B Phase 1	136	85	0.62
Site B Phase 4	157	50	0.32
Site C Phase 1	125	209	1.67
Site C Phase 4	111	52	0.47

Supplemental Table 7

Mean number of immunohistochemistry (IHC) slides per case shown at each of the 3 sites for phase 1 (baseline/no AI) compared to phase 4 (full AI assistance with concurrent-read). The data shows similar patterns of reduction in phase 4 to phase 1 which is supportive of the percentage reduction in cases with IHC requested shown in Table 5.

Supplemental Table 8

Participation in the national UK uropathology external quality assurance scheme (EQA)
On the specialist register for histopathology and regular participation in prostate biopsy reporting
More than 6 months' experience in uropathology post specialist registration
Participation in study-specific Gleason program: completion of Gleason grading benchmarking survey and subsequent consensus meeting that occurred on 8-November-2021 at study launch using EQA cases. The Gleason benchmarking survey showed a baseline Fleiss' kappa level of agreement for GG1 vs GG2-5 between the 7 pathologists at the beginning of the study of 0.63.
In addition, pathologists were strongly encouraged to complete any national or international web-based exercises on Gleason grading where grading has been adjudicated by experts or consensus and to which they have access including ISUP resources.

Supplemental Table 8

Criteria for participating pathologists in the Articulate Pro study

Supplemental Table 9

Potential Technical Obstacle	Mitigation / Observation
**Data Integrity**	WSIs were screened for artefacts (blurring, tissue folds) prior to AI inference.
**System Integration**	Verified compatibility between Paige AI platform and digital pathology and IT infrastructure
**Operational Uptime**	High availability maintained; minor network routing issues resolved via IT support.
**User Access**	Routine credentialing and account reactivations were handled via standard helpdesk workflows.

Supplemental Table 9

A summary of potential technical obstacles and their mitigating actions.

Supplemental Figure 3

Supplemental Figure 3a

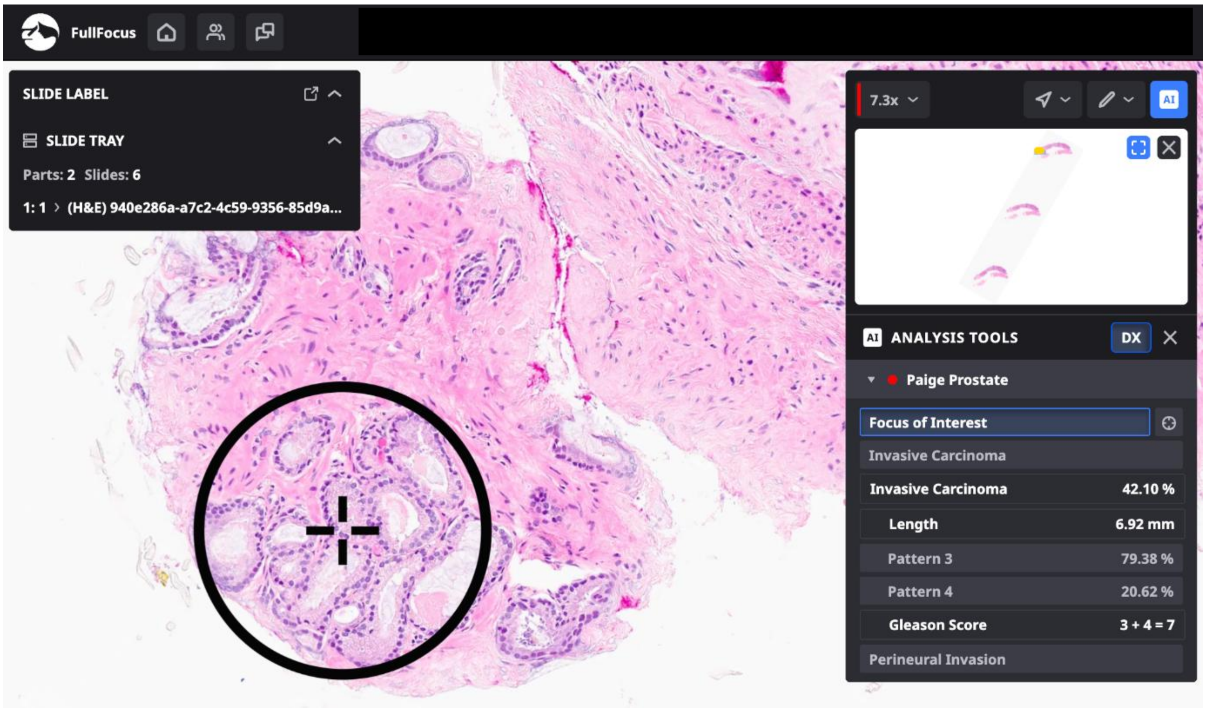

Supplemental Figure 3b

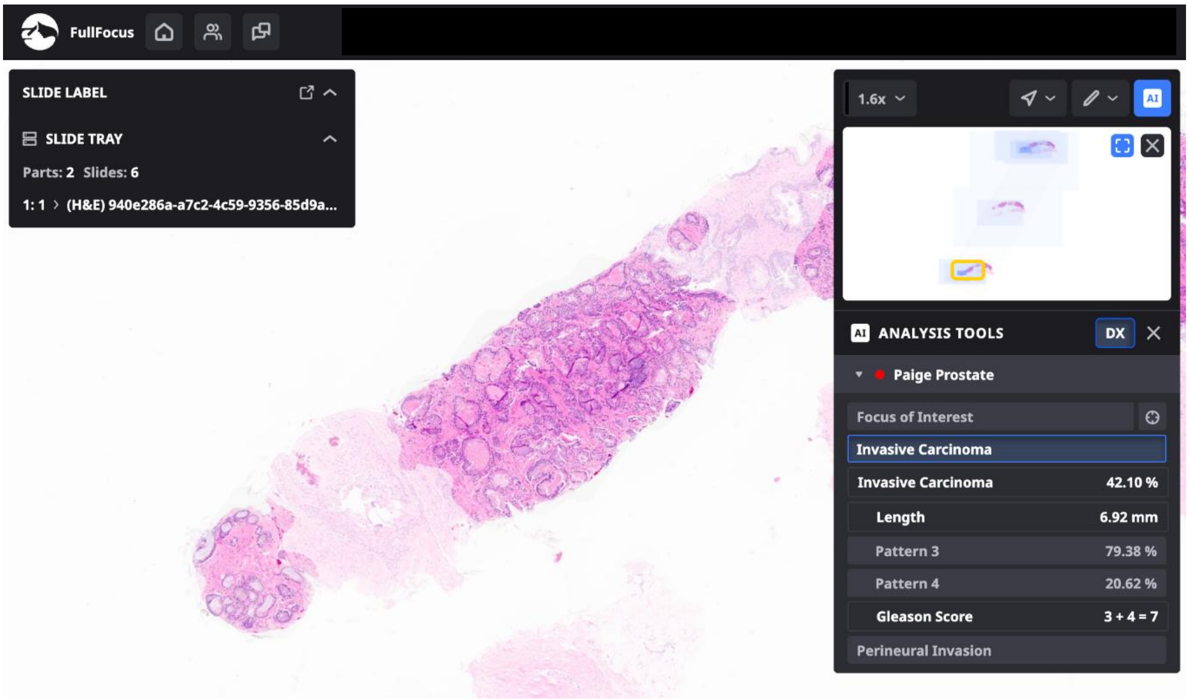

Supplemental Figure 3c

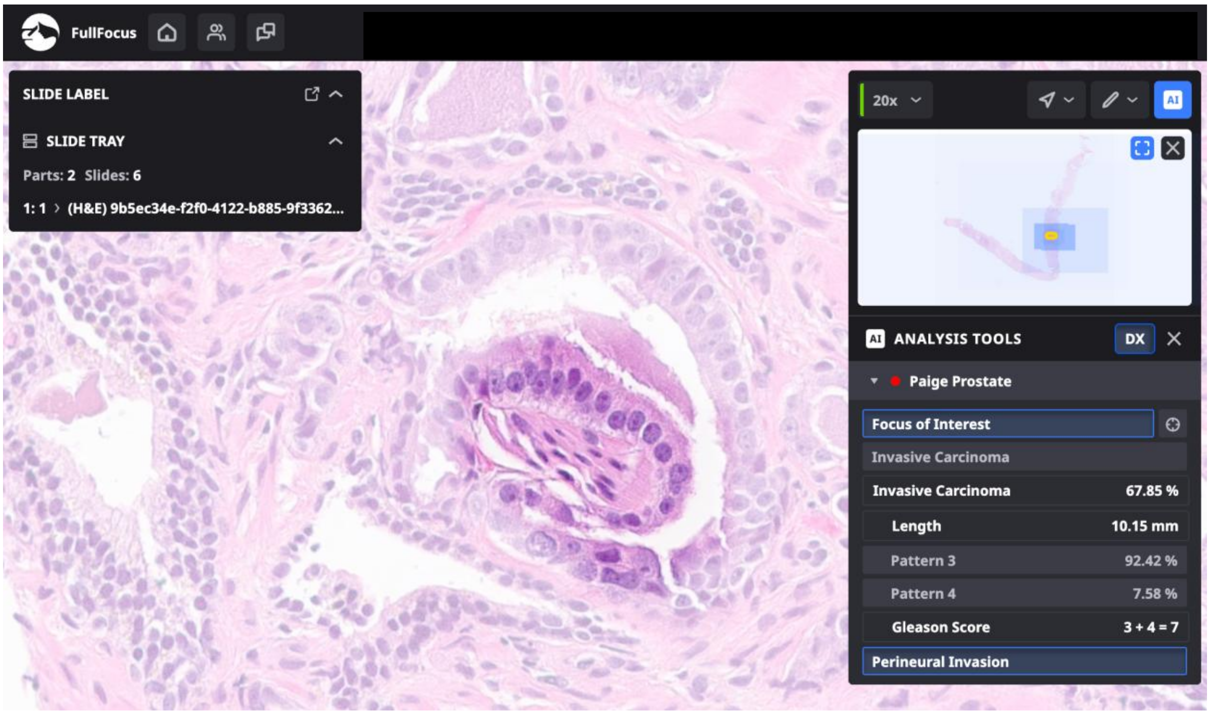

Supplemental Figures 3a-3c

(Please note that fictional patient data is used in these demo slide images)
Figure 3a. Paige Prostate Detect (PaPr Detect) produces a binary prediction for each whole slide image (WSI), either suspicious or not suspicious. A crosshair is presented on suspicious slides, indicating the area with the greatest probability of harbouring cancer.
Figure 3b. PaPr can fog out regions with benign tissue, thus highlighting suspicious areas. In addition, it provides a panel (right) with several outputs, including the percentage of invasive carcinoma, its length, as well as the Gleason patterns present (together with their corresponding percentages). A final Gleason Score is given, and a warning that perineural invasion (PNI) is detected is also shown.
Figure 3c. PaPr highlights a PNI focus by ghosting out the surrounding regions, the viewer's attention is drawn to the focus of interest. In addition, additional information on the tumour is displayed, including tumour percentage, tumour length, Gleason patterns present together with their corresponding percentage, as well as the final Gleason Score predicted.

Supplemental Table 10

Number of core biopsies	No cases phase 1	Mean phase 1	SD phase 1	No cases phase 4	Mean phase 4	SD phase 4	
SITE A	303	17.07	6.29	336	16.11	6.96	t=1.83 p=0.07
SITE B	136	16.63	4.96	156*	15.72	5.32	t=1.50 p=0.13
SITE C	118*	23.47	10.70	108*	21.57	12.11	t=1.3 p=0.2

Supplemental Table 10

The mean number of prostate core biopsies per case shown per site and for phases 1 and 4. *Data on number of core biopsies not available for all site B and C cases; 7/125 cases missing for phase 1 and 3/111 cases missing this data in site C. Data missing for one case in site B in phase 4.

Supplemental Table 11

Details (tissue type/specimen type/preparation/ stain)	Notes
All listed are prostate biopsies<br><br>Benign & inflammation – TRUS	
Benign (levels and single sections)	Suspicious area from PaPr on specimen 4, which seems to be appropriate flag, but not cancer
Acinar adenocarcinoma, Gleason Score 6 (3+3)	Tiny bits of cancer in specimen 1 Gleason 6 (3+3)
Acinar adenocarcinoma, Gleason Score 7 (3+4), small focus	
Acinar adenocarcinoma, Gleason Score 7 (3+4) but difficult grading decision as very limited tumour and on the edge	Specimen 3 has a false positive AI output for tumour detection
Acinar adenocarcinoma, tiny focus Gleason Score 6 (3+3) need confirmation with IHC, borderline ASAP.	
Acinar adenocarcinoma, Gleason Score 7 (4+3) but borderline 3+4.	Specimen 2 (3+4/4+3). Specimen 1 (borderline 3+3/3+4)
Acinar adenocarcinoma, Gleason scoring interesting,	Specimen 2, between 4+3/4+4 plus false positive flag on level (L)3 part 3
Acinar adenocarcinoma, Gleason Score 9+ (borderline 4+5/4+4, some areas PaPr called 3+5)	
Adenocarcinoma with intraductal spread of adenocarcinoma	Specimen 3 (Intra ductal carcinoma), specimen 6, partly invasive ductal
Adenocarcinoma with treatment (radiotherapy or hormone effect)	Hormone therapy
PIN&ASAP	Specimens 1 and 2 (no suspicious areas from AI output)
ASAP	No suspicious areas on L1/2 but identified by AI on L3
BCG related granulomatous prostatitis with necrosis. No suspicious areas detected by AI	
Neuroendocrine small cell carcinoma differentiation	
False positive AI	Paige cancer up to 3+4, pathologist ASAP
False negative AI	Specimen 1. Suspicious area from AI on deeper 3 area of GG1 disease, but not on L3, Deeper1/2 where also present
Out of focus	
Acinar adenocarcinoma, tiny focus Gleason Score 6 (3+3) need confirmation with IHC, borderline ASAP. Good example	Specimen 1 (only on L1)(3+3), part 2 (3+4)
Perineural invasion	

Supplemental Table 11

20 prostate biopsy cases used in the retrospective training set for site A. The examples used were good examples of diagnoses and Gleason Scores together with more difficult or borderline examples. Examples of different AI standalone performance also shown with examples of false negative and false positive outputs. Technically poor examples were also shown and non-acinar adenocarcinoma malignancy - small cell neuroendocrine carcinoma differentiation and ductal type adenocarcinoma. Intraductal adenocarcinoma was also included.
